# Potential Therapeutic Targets of Rehmannia Formulations on Diabetic Nephropathy: A Comparative Network Pharmacology Analysis

**DOI:** 10.3389/fphar.2022.794139

**Published:** 2022-03-21

**Authors:** Kam Wa Chan, Kam Yan Yu, Wai Han Yiu, Rui Xue, Sarah Wing-yan Lok, Hongyu Li, Yixin Zou, Jinyuan Ma, Kar Neng Lai, Sydney Chi-wai Tang

**Affiliations:** Department of Medicine, The University of Hong Kong, Hong Kong SAR, China

**Keywords:** integrative medicine, traditional Chinese medicine, diabetic nephropathy, chronic kidney disease, Rehmannia, mechanism, network pharmacology, TNF

## Abstract

**Background:** Previous retrospective cohorts showed that Rehmannia-6 (R-6, Liu-wei-di-huang-wan) formulations were associated with significant kidney function preservation and mortality reduction among chronic kidney disease patients with diabetes. This study aimed to investigate the potential mechanism of action of common R-6 variations in a clinical protocol for diabetic nephropathy (DN) from a system pharmacology approach.

**Study Design and Methods:** Disease-related genes were retrieved from GeneCards and OMIM by searching “Diabetic Nephropathy” and “Macroalbuminuria”. Variations of R-6 were identified from a published existing clinical practice guideline developed from expert consensus and pilot clinical service program. The chemical compound IDs of each herb were retrieved from TCM-Mesh and PubChem. Drug targets were subsequently revealed via PharmaMapper and UniProtKB. The disease gene interactions were assessed through STRING, and disease–drug protein–protein interaction network was integrated and visualized by Cytoscape. Clusters of disease–drug protein–protein interaction were constructed by Molecular Complex Detection (MCODE) extension. Functional annotation of clusters was analyzed by DAVID and KEGG pathway enrichment. Differences among variations of R-6 were compared. Binding was verified by molecular docking with AutoDock.

**Results:** Three hundred fifty-eight genes related to DN were identified, forming 11 clusters which corresponded to complement and coagulation cascades and signaling pathways of adipocytokine, TNF, HIF-1, and AMPK. Five variations of R-6 were analyzed. Common putative targets of the R-6 variations on DN included ACE, APOE, CCL2, CRP, EDN1, FN1, HGF, ICAM1, IL10, IL1B, IL6, INS, LEP, MMP9, PTGS2, SERPINE1, and TNF, which are related to regulation of nitric oxide biosynthesis, lipid storage, cellular response to lipopolysaccharide, inflammatory response, NF-kappa B transcription factor activity, smooth muscle cell proliferation, blood pressure, cellular response to interleukin-1, angiogenesis, cell proliferation, peptidyl-tyrosine phosphorylation, and protein kinase B signaling. TNF was identified as the seed for the most significant cluster of all R-6 variations. Targets specific to each formulation were identified. The key chemical compounds of R-6 have good binding ability to the putative protein targets.

**Conclusion:** The mechanism of action of R-6 on DN is mostly related to the TNF signaling pathway as a core mechanism, involving amelioration of angiogenesis, fibrosis, inflammation, disease susceptibility, and oxidative stress. The putative targets identified could be validated through clinical trials.

## Introduction

In 2021, 10.5% of the world population were diabetic, with a rising trend (Sun et al., 2021). Of the diabetic patients, 25%–40% develop nephropathy from long-standing diabetes, characterized by glomerular basement membrane thickening, foot process effacement, mesangial expansion, glomerulosclerosis, interstitial fibrosis, and the formation of Kimmelstiel-Wilson nodules (Alsaad and Herzenberg, 2007; Alicic et al., 2017). The pathogenesis of diabetic nephropathy (DN) is multifactorial (Tang and Yiu, 2020) involving the complement cascade (Flyvbjerg, 2017; Yiu et al., 2018), metabolic (Forbes et al., 2003; Goh and Cooper, 2008), immunity (Lin et al., 2012; Pichler et al., 2017; Tang and Yiu, 2020), inflammatory, (Navarro-González et al., 2011; Pichler et al., 2017), oxidative stress (Kashihara et al., 2010; Li et al., 2014a; Sagoo and Gnudi, 2018), coagulation (Madhusudhan et al., 2016; Oe et al., 2016), and hemodynamic (Jerums et al., 2010; Premaratne et al., 2015; Alicic et al., 2017; Tonneijck et al., 2017) pathways, ending with renal fibrosis.

Diabetic kidney disease (DKD) is conventionally diagnosed by the presence of albuminuria, reduced kidney function, and clinical history excluding other etiologies (Alicic et al., 2017; Heerspink et al., 2019). Macroalbuminuria signifies established nephropathy and increases the specificity of DKD diagnosis (Furuichi et al., 2018; Persson and Rossing, 2018). The conventional renin–angiotensin–aldosterone system (RAAS) blockade offers limited effect in reducing end-stage kidney failure and mortality (Hsu et al., 2014; Palmer et al., 2015; Chan and Tang, 2018; Nistor et al., 2018). More regimens targeting other mechanisms are needed. DKD leads the cause of end-stage kidney failure in most regions among other types of chronic kidney disease (CKD). Kidney replacement therapy (dialysis or transplantation) is required once end stage is reached, and the accessibility is often limited (Thomas et al., 2016; Tang et al., 2020; Tang and Yiu, 2020).

Previous registry studies showed that individualized Chinese medicine (CM) treatment was associated with slower decline of kidney function and reduced risk of end-stage kidney failure and mortality among patients with CKD and diabetes (Hsieh et al., 2014; Lin et al., 2015; Huang et al., 2018; Chan et al., 2022). CM formulations are prescribed based on symptom-based diagnosis (Chan et al., 2020a; Chan et al., 2020b; Chan et al., 2021a; Chan et al., 2021b; Chan et al., 2021c; Shu et al., 2021), which predicts renal function decline independent of blood pressure, blood glucose, lipids, and urine albumin control (Chan et al., 2021b). The CM used in these cohorts mostly contained a classical CM formulation, namely, Rehmannia-6 complex (R-6, Liu-wei-di-huang-wan) with variations according to the symptom-based diagnosis. R-6 contains *Rehmanniae Radix* (Di-huang) (Yokozawa et al., 2004), *Corni Fructus* (Shan-zhu-yu) (Gao et al., 2021), *Dioscoreae Rhizoma* (Shan-yao) (Luo, 2008), *Poria* (Fu-ling) (Sun, 2014), *Moutan Cortex* (Mu-dan-pi) (Wang et al., 2017b), and *Alismatis Rhizoma* (Ze-xie) (Zhang et al., 2017a). According to classical CM theory, R-6 replenishes the *kidney* and clears the waste including fluid retention.

Unlike single herbal extracts or synthesized chemical compounds, CM formulations are complex, containing multiple chemicals, and therefore exerts systemic effects through orchestrated pathways in real-world practice (Zhang et al., 2019). Traditional *in vivo* and *in vitro* studies focusing on specific mechanisms may have limitations for the assessment of interaction between CM formulations and disease with multifactorial pathophysiology (Li et al., 2014b; Zhang et al., 2019). Network pharmacology offers a whole-system approach to delineate the possible mechanisms underpinning observed clinical effect based on existing *in vivo* and *in vitro* evidence (Hopkins, 2008; Zhang et al., 2019). This systematic and systemic approach could identify the mechanisms and target proteins of each formulation for subsequent validation in clinical trials.

A protocol with five variations of R-6 was formulated based on previous expert consensus and a pilot service program and is undergoing clinical trial (Chan et al., 2016). R-6 was modified based on the phenotypes of patients according to the traditional CM theory. There are limited system pharmacology data regarding the putative mechanism of these R-6 variations on DN. This study aims to investigate the potential mechanism of the therapeutic action of common R-6 variations for DN. We retrieved the genes and potential targets of DN, the chemical compounds of CMs, and the corresponding targets of R-6 through data mining. A drug–disease network was then constructed with pathway analysis to delineate the interaction between R-6 and DN. Through comparing the pathways expected to act on by different R-6 variations, we identified the putative targets of R-6 on DN and the potential differences among different variations.

## Materials and Methods

### Genes and Potential Targets Related to DN

**FIGURE 1:**
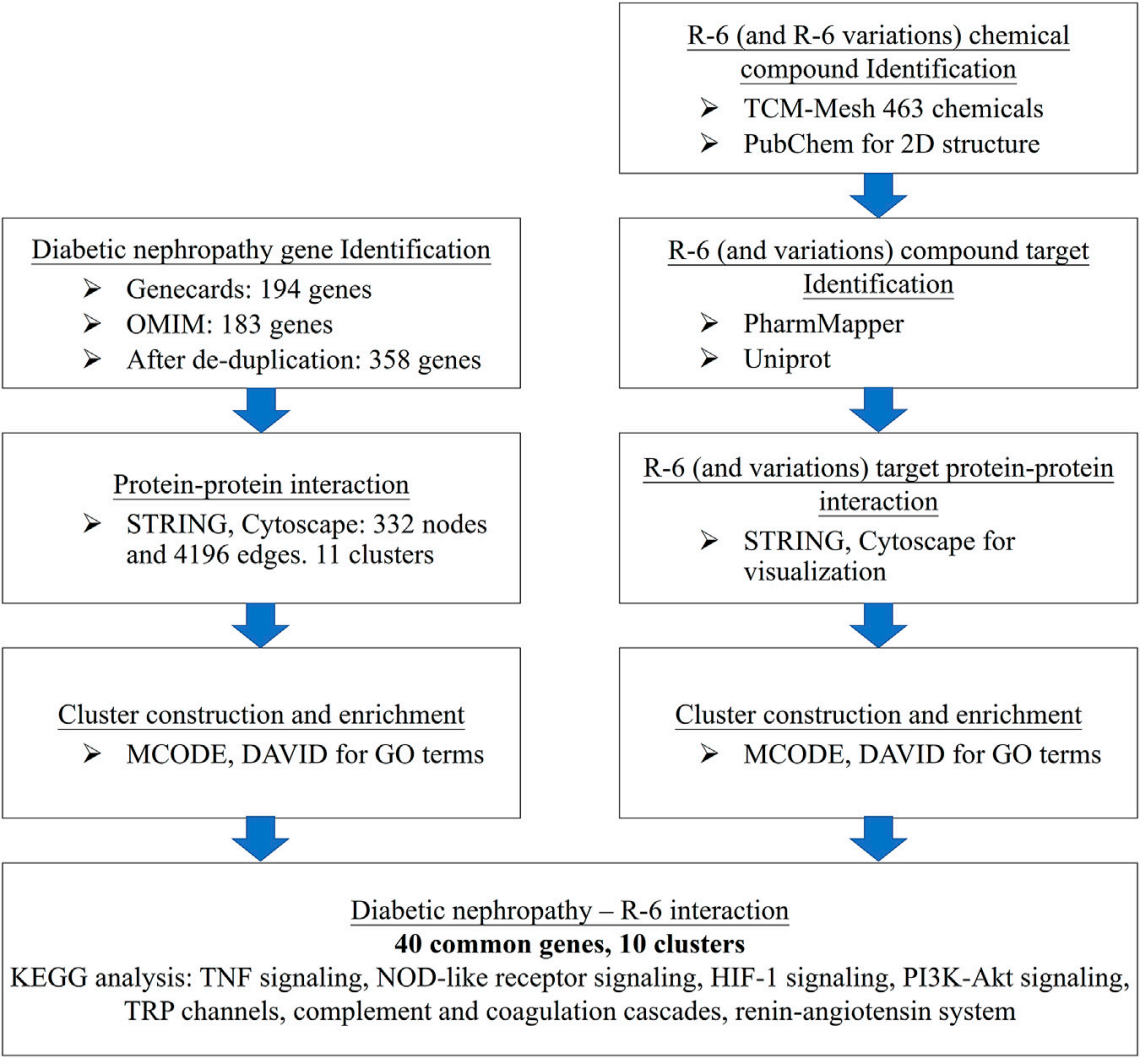
Computational workflow of disease–drug network analysis. Disease-related genes were retrieved from GeneCards and OMIM. The chemical compound IDs of each herb were retrieved from TCM-Mesh and PubChem. Drug targets were revealed via PharmaMapper and UniProtKB. The disease gene interactions were assessed through STRING, and the disease–drug protein–protein interaction network was integrated and visualized by Cytoscape. Functional annotation of clusters was analyzed by DAVID and KEGG pathway enrichment.

The computational workflow is summarized in Figure 1. First, disease-related genes were retrieved from GeneCards (https://www.genecards.org/) (Stelzer et al., 2016) and the Online Mendelian Inheritance in Man (OMIM) (https://www.omim.org/) (Hamosh et al., 2000). GeneCards, operated by The Weizmann Institute of Science and LifeMap Sciences, provides richly annotated disease genetics integrated from over 150 web sources. OMIM provides genotypic and phenotypic information on molecular contributions in human genetic disorders. We searched the databases with keywords “Diabetic Nephropathy” and “Macroalbuminuria” as macroalbuminuria signifies the development of nephropathy and increases the specificity of DN (Tang and Yiu, 2020) and is commonly included as a recruitment criterion of diabetic kidney disease-related clinical trials (Heerspink et al., 2019; Perkovic et al., 2019). The genetic datasets from GeneCards and OMIM were integrated for a more comprehensive analysis (Supplementary Table S1).

### Compounds of R-6 and Their Potential Targets

The variations of R-6 were identified from an existing clinical practice guideline developed based on expert consensus in mainland China (Zhang, 2010; Chan, 2018), which is undergoing a randomized clinical trial for evaluation in Hong Kong (Chan et al., 2016; Chan et al., 2019). The clinical guideline is compatible with the prescription pattern of previously reported big-data studies from Taiwan (Hsieh et al., 2014; Lin et al., 2015; Huang et al., 2018; Guo et al., 2021b) and a pilot service program in Hong Kong (Chan, 2018; Chan et al., 2022).

TCM-Mesh (http://mesh.tcm.microbioinformatics.org/) (Zhang et al., 2019; Zhang et al., 2017b), with data on chemicals, genes, and disease association of more than 6,000 herbs, was used to collect the compound IDs of chemicals in each herb. A total of 463 chemicals were identified including flavonoids, phenolic acids, and alkaloids (Supplementary Table S2). Compound IDs were identified by PubChem (https://pubchem.ncbi.nlm.nih.gov/) (Kim et al., 2019), a freely accessible chemistry database maintained by the National Institutes of Health (NIH), where we acquired the 2D structures of each chemical in sdf format files. sdf format files were then imported into an open web server, PharmaMapper (http://www.lilab-ecust.cn/pharmmapper/) (Wang et al., 2017a), to identify human drug targets of chemicals. UniProtKB (https://www.uniprot.org/) (The UniProt Consortium, 2019), a central hub that integrates the functional annotations of proteins, was applied to convert the unstandardized protein naming into the official symbol by selecting “Homo sapiens”.

### Disease–Drug Network Construction

Both the integrated disease and drug targets were imported into the Search Tool for the Retrieval of Interacting Genes/Proteins (STRING) (https://string-db.org/, version 11.0) (Szklarczyk et al., 2019) to assess and visualize the protein–protein interactions between disease and drug mechanisms at the molecular level to identify potential targets of R-6 on DN. The returned interaction data were expressed with a network visualization software, namely, Cytoscape (http://cytoscape.org/, version 3.7.2) (Shannon et al., 2003), to integrate the complex network of molecular interactions. To explore relevant clusters with extensive protein interactions, we used the Molecular Complex Detection (MCODE), a plug-in of Cytoscape, to identify clusters (densely interconnected regions) in the network. Each cluster represents an independent network of biological mechanisms. The disease, drug, and disease–drug protein–protein interaction networks and clusters were constructed for DN, R-6, and variations of R-6.

### Gene Ontology and Pathway Enrichment Analysis

Each cluster returned by MCODE was imported into the Database for Annotation, Visualization and Integrated Discovery (DAVID) (https://david.ncifcrf.gov/summary.jsp, version 6.8) (Huang et al., 2009), a web server which integrates the functional annotations of a large set of genes with reference to disease association, biological pathways from various databases for a more profound understanding of disease pathogenesis, and identification of potential targets for treatment. All functional annotation data were corrected by the Bonferroni method to minimize type 1 error arising from multiple comparisons. All targets of DN and R-6 variations and the top cluster of the disease–gene interaction were further analyzed and compared by the Kyoto Encyclopedia of Genes and Genomes (KEGG) pathway enrichment.

### Validation by Molecular Docking

The chemical structure of key compounds of the CMs of R-6 was extracted from PubChem as mol2 files and processed in pdbqt format in AutoDockTools 1.5.6 for binding analysis. The crystal structures of the putative gene/protein targets obtained from the network and pathway analyses were extracted from the RCSB Protein Data Bank (https://www.rcsb.org/). Water molecules and inactive ligands were removed before the proteins were hydrogenated and charged (Guo et al., 2021a). A blind docking strategy was used to screen through each protein for all possible binding sites with a grid size of 126 × 126× 126 points. Free energy of binding (in kcal/mol) were obtained from AutoDock Vina as the indicator of the binding likelihood (Lee et al., 2012; Cournia et al., 2017; Heinzelmann and Gilson, 2021). Negative values of free energy indicate possible binding in simulation. More negative scoring indicates stronger binding affinity.

## Results

### DN

**FIGURE 2:**
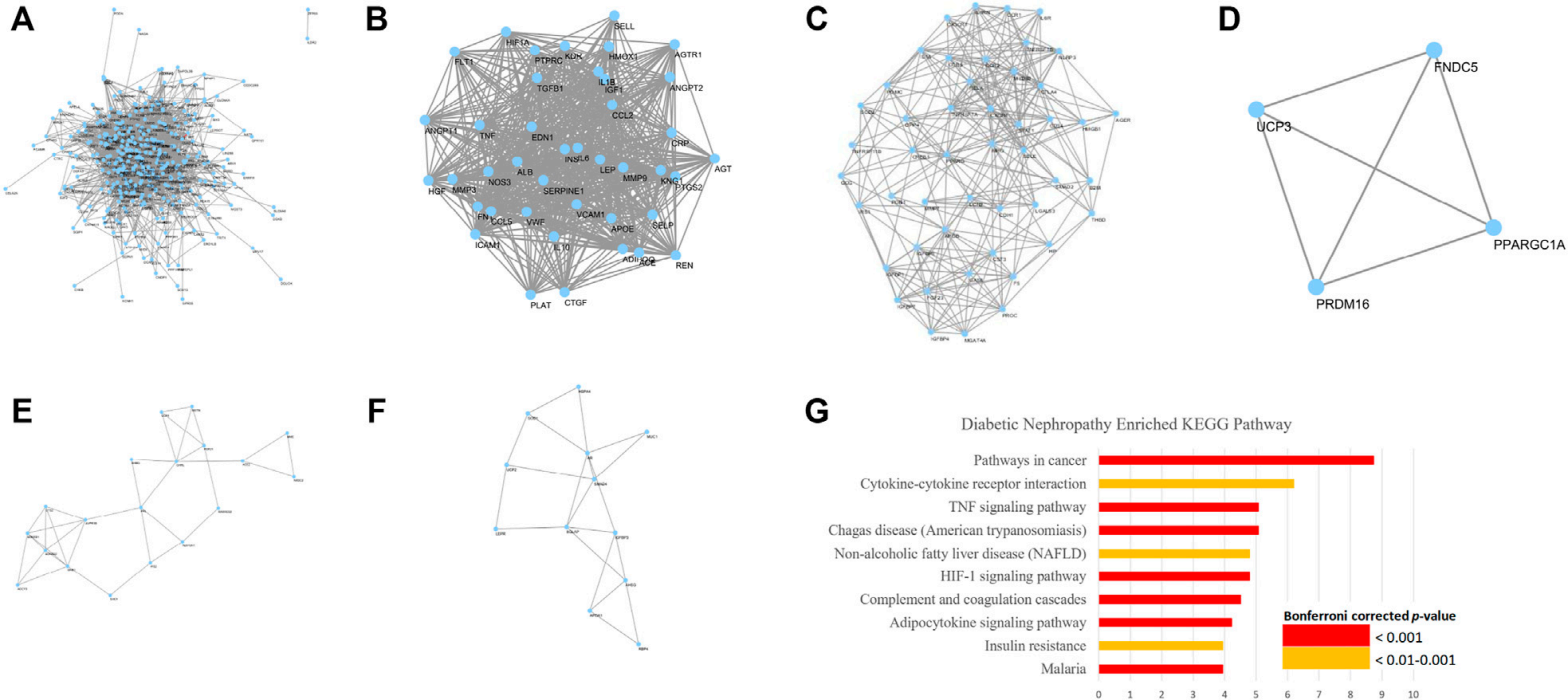
Network and representative clusters of DN. Key pathogenesis of DN includes the complement and coagulation cascades, adipocytokine signaling pathway, TNF signaling pathway, HIF-1 signaling pathway, and AMPK signaling pathway. **(A)** A total of 9,621 pairs of genes were identified from GeneCards and OMIM. **(B**–**F)** Major Cluster of Genes Related to Diabetic Nephropathy and **(G)** KEGG enrichment analysis on the DN pathogenesis

A total of 358 DN-related genes were obtained from GeneCards and OMIM (Figure 2A). The network contains 332 nodes and 4,196 edges. Eleven clusters (Figures 2B–F) were identified from the analysis by MCODE. Gene ontology analysis showed that these genes corresponded to 71 ontology terms. The top 20 most significant terms included the response to hypoxia (GO: 0001666), inflammatory response (GO: 0006954), glucose homeostasis (GO: 0042593), response to drug (GO: 0042493), positive regulation of angiogenesis (GO: 0045766), regulation of blood pressure (GO: 0008217), leukocyte migration (GO: 0050900), positive regulation of smooth muscle cell proliferation (GO: 0048661), response to activity (GO: 0014823), positive regulation of cytosolic calcium ion concentration (GO: 0007204), acute-phase response (GO: 0006953), platelet degranulation (GO: 0002576), positive regulation of gene expression (GO: 0010628), positive regulation of nitric oxide biosynthetic process (GO: 0045429), aging (GO: 0007568), response to glucocorticoid (GO: 0051384), positive regulation of transcription from RNA polymerase II promoter (GO: 0045944), cellular response to lipopolysaccharide (GO: 0071222), negative regulation of apoptotic process (GO: 0043066), and response to mechanical stimulus (GO: 0009612) (Supplementary Datasheet S3).

Further KEGG pathway analysis showed that the DN-related pathways were closely related to the complement and coagulation cascades, adipocytokine signaling pathway, TNF signaling pathway, HIF-1 signaling pathway, and AMPK signaling pathway, which share similar mechanisms with insulin resistance, rheumatoid arthritis, non-alcoholic fatty liver disease, and various infectious diseases and cancers (Figure 2G; Supplementary Datasheet S4).

### Putative Targets of R-6

**FIGURE 3:**
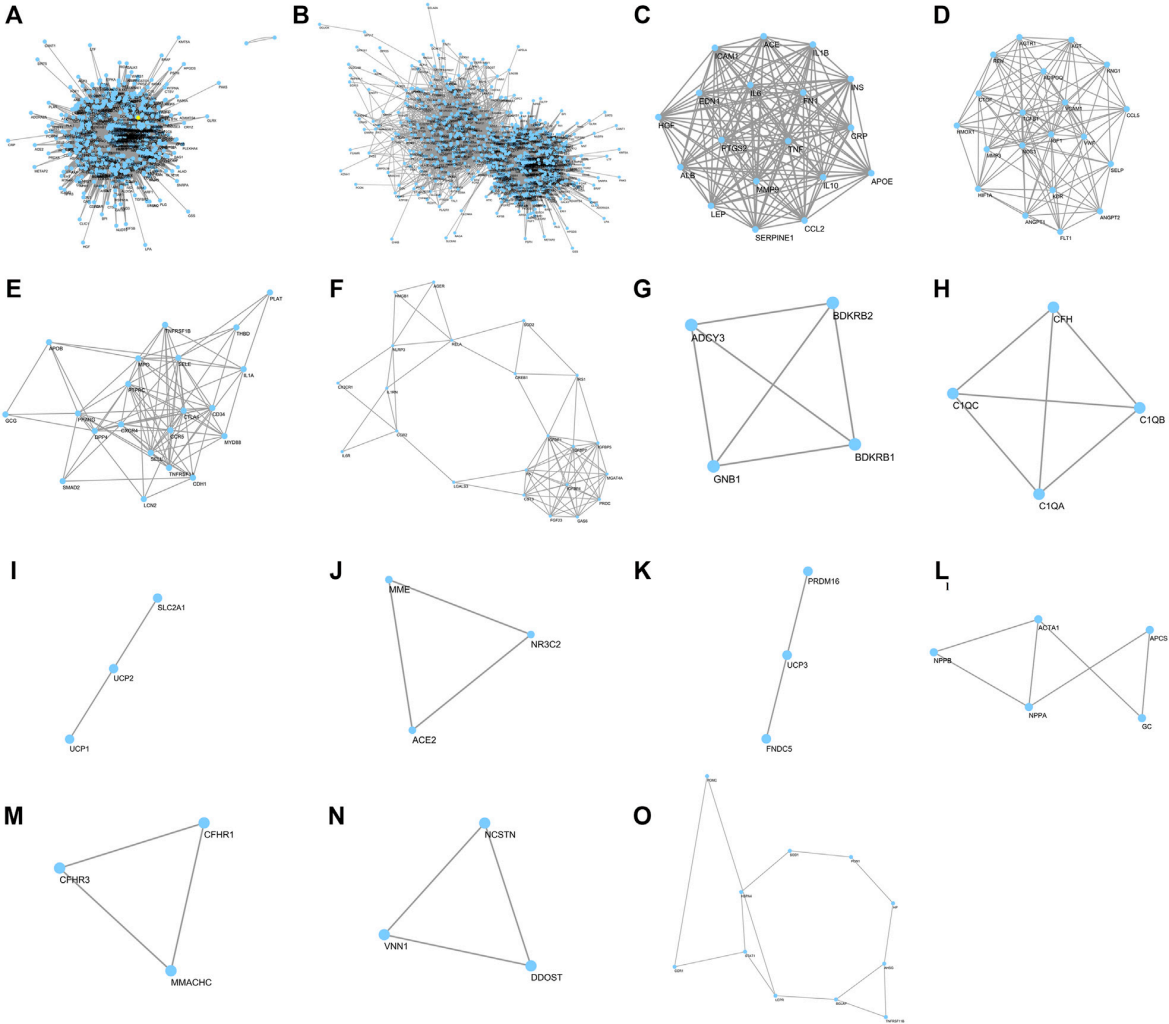
Network of R-6 putative targets and clusters of DN–R-6 interaction network. Forty DN-related genes were overlapped with and R-6; **(A)** 449 potential gene targets of R-6; **(B)** putative targets of R-6 on diabetic DN; **(C**–**O)** Major clusters of putative targets of R-6 on DN

R-6 is a formulation containing *Rehmanniae Radix*, *Corni Fructus*, *Dioscoreae Rhizoma*, *Poria*, *Moutan Cortex*, and *Alismatis Rhizoma* (Table 1) and contains 64 identifiable chemical compounds (Supplementary Table S2) (out of the 463 chemicals from all R-6 variations). Four hundred forty-nine potential gene targets of R-6 were identified (Figure 3A). The top 20 most significant terms included steroid hormone-mediated signaling pathway (GO: 0043401), peptidyl-tyrosine autophosphorylation (GO: 0038083), transcription initiation from RNA polymerase II promoter (GO: 0006367), negative regulation of apoptotic process (GO: 0043066), proteolysis (GO: 0006508), protein autophosphorylation (GO: 0046777), extracellular matrix disassembly (GO: 0022617), peptidyl-tyrosine phosphorylation (GO: 0018108), response to hypoxia (GO: 0001666), oxidation-reduction process (GO: 0055114), response to drug (GO: 0042493), positive regulation of phosphatidylinositol 3-kinase signaling (GO: 0014068), protein phosphorylation (GO: 0006468), cellular response to insulin stimulus (GO: 0032869), glutathione metabolic process (GO: 0006749), phosphatidylinositol-mediated signaling (GO: 0048015), collagen catabolic process (GO: 0030574), response to estrogen (GO: 0043627), leukocyte migration (GO: 0050900), and purine-containing compound salvage (GO: 0043101) (Supplementary Data S5).

**TABLE 1:** Variations of R-6.

**R-6**	**Formulation 1**	**Formulation 2**	**Formulation 3**	**Formulation 4**	**Formulation 5**
*Rehmanniae Radix* (Di-huang)	*Rehmanniae Radix* (Di-huang)	*Rehmanniae Radix* (Di-huang)	*Rehmanniae Radix* (Di-huang)	*Rehmanniae Radix* (Di-huang)	*Rehmanniae Radix* (Di-huang)
*Dioscoreae Rhizoma* (Shan-yao)	*Dioscoreae Rhizoma* (Shan-yao)	*Dioscoreae Rhizoma* (Shan-yao)	*Dioscoreae Rhizoma* (Shan-yao)	*Dioscoreae Rhizoma* (Shan-yao)	*Dioscoreae Rhizoma* (Shan-yao)
*Corni Fructus* (Shan-zhu-yu)	*Corni Fructus* (Shan-zhu-yu)	*Corni Fructus* (Shan-zhu-yu)	*Corni Fructus* (Shan-zhu-yu)	*Corni Fructus* (Shan-zhu-yu)	*Corni Fructus* (Shan-zhu-yu)
*Moutan Cortex* (Mu-dan-pi)	*Moutan Cortex* (Mu-dan-pi)	*Moutan Cortex* (Mu-dan-pi)	*Moutan Cortex* (Mu-dan-pi)	*Moutan Cortex* (Mu-dan-pi)	*Moutan Cortex* (Mu-dan-pi)
*Poria* (Fu-ling)	*Poria* (Fu-ling)	*Poria* (Fu-ling)	*Poria* (Fu-ling)	*Poria* (Fu-ling)	*Poria* (Fu-ling)
*Alismatis Rhizoma* (Ze-xie)	*Alismatis Rhizoma* (Ze-xie)	*Alismatis Rhizoma* (Ze-xie)	*Ginseng Radix et Rhizoma* (Ren-shen)	*Alismatis Rhizoma* (Ze-xie)	*Alismatis Rhizoma* (Ze-xie)
	*Aucklandiae Radix* (Mu-xiang)	*Chaenomelis Fructus* (Mu-gua)	*Astragali Radix* (Huang-qi)	*Ligustri Lucidi Fructus* (Nv-zhen-zi)	*Cinnamomi Ramulus* (Gui-zhi)
	*Amomi Fructus* (Sha-ren)	*Aucklandiae Radix* (Mu-xiang)	*Zingiberis Rhizoma Recens* (Sheng-jiang)	*Eclipta prostrata* (Mo-Han-Lian)	*Aconiti Lateralis Radix Praeparata* (Fu-zi)
	*Citri Reticulatae Pericarpium* (Chen pi)	*Arecae Semen* (Bing-lang)	*Jujubae Fructus* (Da-zao)		*Ligustri Lucidi Fructus* (Nv-zhen-zi)
	*Ginseng Radix et Rhizoma* (Ren-shen)	*Aconiti Lateralis Radix Praeparata* (Fu-zi)			
	*Pinelliae Rhizoma* (Ban-xia)	*Zingiberis Rhizoma* (Gan-jiang)			
	*Atractylodis Macrocephalae Rhizoma* (Bai-zhu)	*Glycyrrhizae Radix et Rhizoma* (Gan-cao)			
	*Glycyrrhizae Radix et Rhizoma* (Gan-cao)	*Zingiberis Rhizoma Recens* (Sheng-jiang)			
		*Jujubae Fructus* (Da-zao)			
		*Tsaoko Fructus* (Cao-guo)			

Rehmannia-6 complex (R-6, Liu-wei-di-huang-wan) was the most used Chinese medicine formulation in the cohorts showing beneficial effect on diabetic kidney disease. Variations of R-6 were identified from a published existing clinical practice guideline developed from expert consensus and pilot clinical service program, which is undergoing a randomized clinical trial for evaluation.

KEGG pathway analysis showed that the R-6-related pathways involve complement and coagulation cascades, VEGF signaling pathway, HIF-1 signaling pathway, T-cell and B-cell receptor signaling pathway, Fc epsilon RI signaling pathway, PI3K-Akt signaling pathway, MAPK signaling pathway, Ras signaling pathway, ErbB signaling pathway, FoxO signaling pathway, PPAR signaling pathway, sphingolipid signaling pathway, Rap1 signaling pathway, neurotrophin signaling pathway, prolactin, estrogen and thyroid hormone signaling pathway, metabolic pathways, insulin signaling pathway, and purine metabolism, sharing similar mechanisms with various infectious diseases and cancers (Supplementary Data S6).

### Putative Targets of R-6 on DN

Forty DN-related genes were overlapped with R-6 (Figure 3B–O; Table 2). Gene ontology analysis on the most significant cluster showed that the putative targets of R-6 on DN are strongly related to 44 ontology terms in 10 clusters, which included positive regulation of nitric oxide biosynthetic process (GO: 0045429), negative regulation of lipid storage (GO: 0010888), cellular response to lipopolysaccharide (GO: 0071222), inflammatory response (GO: 0006954), positive regulation of NF-kappa B transcription factor activity (GO: 0051092), positive regulation of smooth muscle cell proliferation (GO: 0048661), regulation of blood pressure (GO: 0008217), response to glucocorticoid (GO: 0051384), cellular response to interleukin-1 (GO: 0071347), angiogenesis (GO: 0001525), and negative regulation of blood coagulation (GO: 0030195) in cluster 1; positive regulation of phosphatidylinositol 3-kinase signaling (GO: 0014068), angiogenesis (GO: 0001525), positive regulation of cellular protein metabolic process (GO: 0032270), response to hypoxia (GO: 0001666), positive regulation of peptidyl-tyrosine phosphorylation (GO: 0050731), platelet degranulation (GO: 0002576), renin–angiotensin regulation of aldosterone production (GO: 0002018), positive regulation of ERK1 and ERK2 cascade (GO: 0070374), positive regulation of cell migration (GO: 0030335), regulation of blood pressure (GO: 0008217), and response to glucose (GO: 0009749) in cluster 2; inflammatory response (GO: 0006954), leukocyte migration (GO: 0050900), and response to lipopolysaccharide (GO: 0032496) in cluster 3; positive regulation of dendritic cell differentiation (GO: 2001200) and inflammatory response (GO: 0006954) in cluster 4; positive regulation of cytosolic calcium ion concentration (GO: 0007204) in cluster 5; complement activation (GO: 0006956), innate immune response (GO: 0045087), and proteolysis (GO: 0006508) in cluster 6; mitochondrial transport (GO: 0006839) in cluster 7; angiotensin maturation (GO: 0002003) in cluster 8; positive regulation of brown fat cell differentiation (GO: 0090336) in cluster 9; and response to hydrogen peroxide (GO: 0042542) in cluster 13 (Supplementary Data S7).

**TABLE 2:** List of common proteins between diabetic nephropathy and R-6 variations.

	Common target proteins
Between DN and R-6	ACADM, ACE, AKR1B1, ALB, AR, DPP4, F7, GC, HMOX1, HSD11B1, IGF1, KDR, LCK, LCN2, MME, MMP3, MMP7, MMP9, NR3C2, PPARG, RBP4, REN, SHBG, SOD2, SORD, VDR, APCS, CCL5, HGF, LGALS3, NOS3, PADI4, PARP1, REG1A, SELE, SELP, STAT1, PLAT, ACE2, and CRP
Between DN and R-6 variations	ACE, APOE, CCL2, CRP, EDN1, FN1, HGF, ICAM1, IL10, IL1B, IL6, INS, LEP, MMP9, PTGS2, SERPINE1, and TNF^a^

a TNF was identified as the seed for the most significant cluster of all variations of R-6.

**FIGURE 4:**
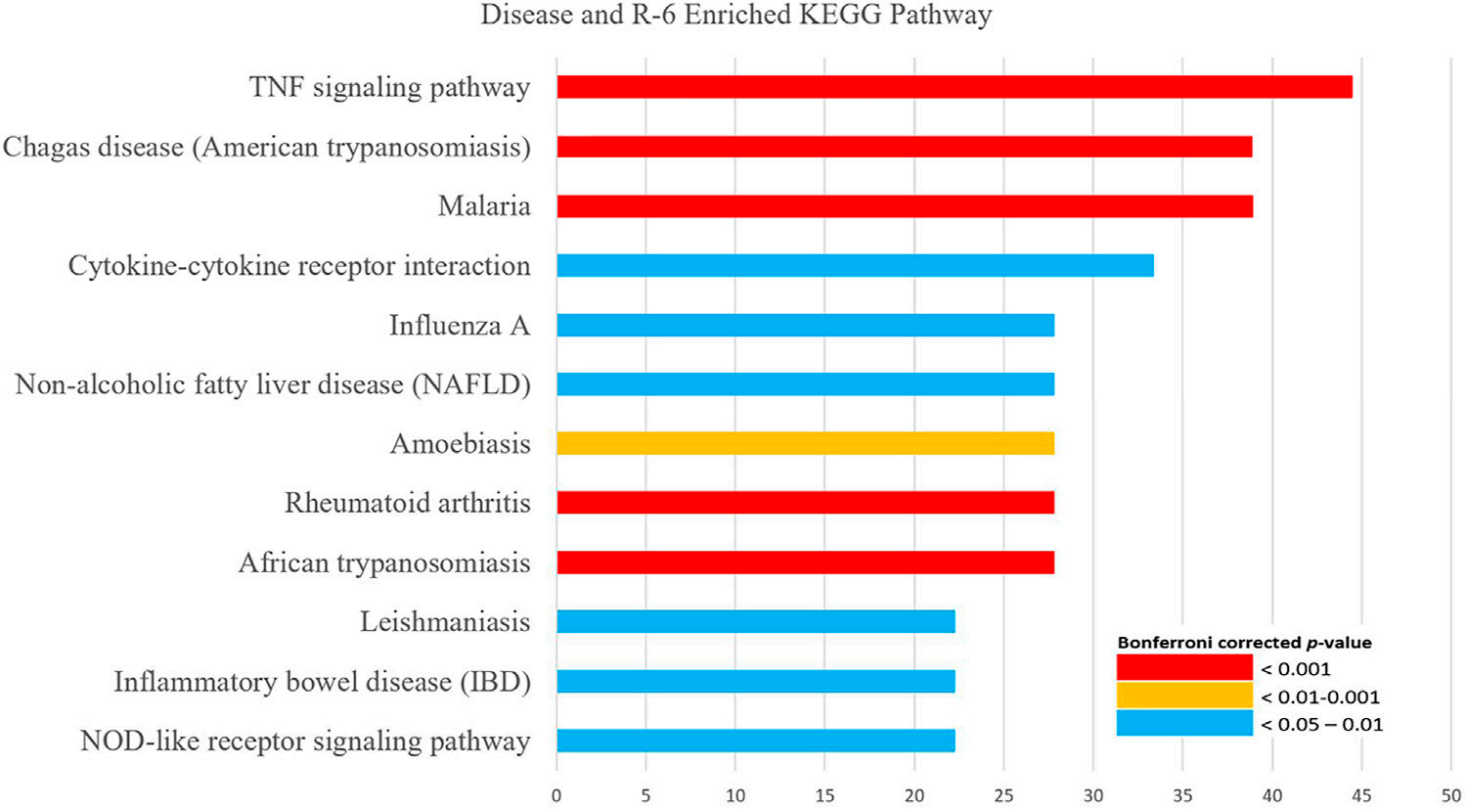
KEGG pathway enrichment analysis of the interaction between DN and R-6. KEGG pathway analysis showed that the putative targets of R-6 on diabetic nephropathy are related to the TNF signaling pathway, NOD-like receptor signaling pathway, HIF-1 signaling pathway, PI3K-Akt signaling pathway, inflammatory mediator regulation of TRP channels, complement and coagulation cascades, and renin–angiotensin system.

KEGG pathway analysis showed that the possible mechanisms of R-6 on DN are related to the TNF signaling pathway, NOD-like receptor signaling pathway, HIF-1 signaling pathway, PI3K-Akt signaling pathway, inflammatory mediator regulation of TRP channels, complement and coagulation cascades, and renin–angiotensin system, which share similar mechanisms with non-alcoholic fatty liver disease, inflammatory bowel disease, rheumatoid arthritis, and various types of infectious diseases and cancers (Figure 4; Supplementary Data S8).

### Comparative Analysis on the Putative Targets of R-6 Variations on DN

Five variations of R-6 were identified from an existing clinical practice guideline and were analyzed (Table 1). The common putative targets of the R-6 variations on DN included ACE, APOE, CCL2, CRP, EDN1, FN1, HGF, ICAM1, IL10, IL1B, IL6, INS, LEP, MMP9, PTGS2, SERPINE1, and TNF. TNF was identified as the seed for the most significant cluster of all R-6 variations. These common targets were related to the TNF signaling pathway, NOD-like receptor signaling pathway, HIF-1 signaling pathway, PI3K-Akt signaling pathway, inflammatory mediator regulation of TRP channels, complement and coagulation cascades, and renin–angiotensin system, which share similar mechanisms with rheumatoid arthritis, systemic lupus erythematosus, non-alcoholic fatty liver disease, inflammatory bowel disease, and various types of infectious diseases and cancers.

Formulation 1, 3, and 4 had 9, 10, and 7 clusters with significant interactions with DN, respectively. These clusters involved positive regulation of the nitric oxide biosynthetic process (GO: 0045429), negative regulation of lipid storage (GO: 0010888), cellular response to lipopolysaccharide (GO: 0071222), and inflammatory response (GO: 0006954) and positive regulation of NF-kappa B transcription factor activity (GO: 0051092) in cluster 1; positive regulation of phosphatidylinositol 3-kinase signaling (GO: 0014068), angiogenesis (GO: 0001525), positive regulation of cellular protein metabolic process (GO: 0032270), response to hypoxia (GO: 0001666), positive regulation of peptidyl-tyrosine phosphorylation (GO: 0050731), platelet degranulation (GO: 0002576), and renin–angiotensin regulation of aldosterone production (GO: 0002018) in cluster 2; and inflammatory response (GO: 0006954) and leukocyte migration (GO: 0050900) in cluster 3, which was highly similar to R-6. Both formulations 1 and 4 also had a cluster with significant interaction on complement activation (GO: 0006956).

There were six clusters with a significant function between formulation 2 and DN. The major cluster of interaction involved regulation of blood pressure (GO: 0008217), positive regulation of ERK1 and ERK2 cascade (GO: 0070374), positive regulation of cell proliferation (GO: 0008284), positive regulation of cell migration (GO: 0030335), positive regulation of T-cell proliferation (GO: 0042102), negative regulation of the endothelial cell apoptotic process (GO: 2000352), cellular response to interleukin-1 (GO: 0071347), negative regulation of the extrinsic apoptotic signaling pathway via death domain receptors (GO: 1902042), positive regulation of MAPK cascade (GO: 0043410), positive regulation of protein kinase B signaling (GO: 0051897), aging (GO: 0007568), cellular response to tumor necrosis factor (GO: 0071356), and extracellular matrix organization (GO: 0030198) in addition to that of R-6 and DN alone.

Formulation 5 formed eight clusters with significant interactions with DN. The two clusters with the most significant interactions included positive regulation of phosphatidylinositol 3-kinase signaling (GO: 0014068), angiogenesis (GO: 0001525), positive regulation of cellular protein metabolic process (GO: 0032270), response to hypoxia (GO: 0001666), positive regulation of peptidyl-tyrosine phosphorylation (GO: 0050731), platelet degranulation (GO: 0002576), and renin–angiotensin regulation of aldosterone production (GO: 0002018) in cluster 2; inflammatory response (GO: 0006954) and leukocyte migration (GO: 0050900) in cluster 3; and complement activation (GO: 0006956) in cluster 6 (Supplementary Data S9).

KEGG analysis showed that formulations 1, 3, and 5 involved the TNF signaling pathway, NOD-like receptor signaling pathway, non-alcoholic fatty liver disease, and inflammatory bowel disease, and formulations 2 and 4 involved inflammatory mediator regulation of TRP channels in addition to the shared pathways (Supplementary Data S10).

### Validation of Binding

From the molecular docking, the key chemical constituents from R-6 have good binding affinity to APOE, CCL2, EDN1, FN1, HGF, ICAM1, IL10, IL1B, IL6, INS, LEP, MMP9, PTGS2, SERPINE1, TNF, and TNF receptor 1, which supported the mechanistic involvement of these related pathways in the R-6’s effect on DN. ACE, CRP, PTGS, and TNF receptor 2 were not assessable due to the large crystal structure (Table 3).

**TABLE 3:** Binding affinity of key chemical compounds of R-6 to putative targets.

Drug	Key chemical constituents	TNFR1	TNF	SERPINE1	MMP9	INS	IL6	IL10	ICAM1	HGF	FN1	EDN1	APOE	IL1B	CCL2	LEP
*Alismatis Rhizoma*	alisol a	−4.73	−2.85	−5.93	−4.51	−5.04	−5.75	−6.2	−3.77	−5.21	−5.24	−5.87	−3.35	−6.12	−3.98	−5.26
	alisol b	−5.26	−4.44	−4.65	−5.93	−6.01	−7.05	−6.77	−3.82	−5.89	−6.02	−5.13	−3.42	−6.97	−4.55	−7.71
	alisol c	−6.28	−3.22	−7.97	−3.29	−7.61	−7.1	−6.29	−3.61	−6.04	−4.78	−5.62	−2.89	−7.47	−5.4	−6.37
*Corni Fructus*	3,6-digalloylglucose	1.98	2.44	2.13	2.57	-2.51	-0.83	4.77	0.05	0.52	0.54	1.69	2.28	2.73	1.51	−2.34
	7,8-dehydropenstemoside	−1.99	−0.56	−1.12	−2.32	−1.68	−2.18	−1.6	−0.14	−2.07	−1.42	−3.73	0.06	−1.65	−1.89	−4.69
	7-*O*-methyl morroniside	−2.93	−2.63	−1.89	−3.87	−3.67	−4.05	−2.15	−1.28	−3.33	−3.81	−4.08	−2.01	−1.95	−2.05	−4.02
*Dioscoreae Rhizoma*	campesterol	−6.47	-4.04	−5.85	−5.76	−6.48	−6.84	−6.03	−5.32	−6.53	−2.01	−6.05	−4.29	−5.74	−5.08	−6.19
	deltoside	−4.3	−2.97	−4.5	−4.79	−4.58	−5.28	−3.83	−3.07	−3.76	−4.41	−5.23	−3.97	−3.8	−3.53	−5.15
	dioscin	−1.25	1.07	−1.29	−4.37	−3.74	-4.03	−4.27	−1.49	−4.73	−2.89	−3.95	4.47	−1.71	−2.25	−3.53
*Moutan Cortex*	benzoylpaeoniflorin	−2.84	−0.6	−1.2	−0.39	−5.41	−3.45	0.36	0.29	−2.62	−2.16	−1.47	1.37	0.59	−1.76	−3.85
	paeoniflorin	−1.12	−0.08	−3.63	−0.94	−4.06	−4.53	−1.63	−0.82	−2.52	−4.01	−3.76	−0.79	−4.7	−2.39	−2.83
	suffruticoside a	−0.86	0.15	−2.79	−2.4	−3.67	−3.46	−0.34	−0.87	−2.01	−2.7	−3.13	−0.24	−0.31	−0.55	−2.32
*Poria*	20-hexadecanoylingenol	1.65	−1.12	−0.78	−1.77	−3.68	−1.41	−3.41	1.59	−5.72	−1.98	−3.53	1.49	−3.98	−1.63	−3.75
	adenine	−3.08	−2.46	−4.23	−3.14	−3.8	−4.46	−3.65	−2.61	−3.47	−4.34	−4.92	−3.75	−4.23	−3.16	−4.49
	beta-amyrin acetate	−6.9	−6.22	−6.89	−5.76	−7.77	−7.42	−8.49	−6.29	−7.71	−6.91	−6.95	−6.5	−8.34	−7.34	−8.44
	*p*-hydroxybenzyl alcohol	−3.51	−3.12	−3.38	−4.4	−3.84	−3.66	−3.01	−3.16	−3.62	−3.39	−3.45	−2.16	−3.73	−3.15	−4.09
*Rehmanniae Radix*	acteoside	5.97	5.97	0.24	1.65	0.22	−0.9	−1.53	5.96	1.94	3.07	0.57	5.97	0.37	3.89	0.64
	catalpol	0.04	−0.25	−2.49	−1.89	−1.18	−2.77	−1.28	−0.79	−2.74	−1.62	−1.92	−0.25	−3.1	−1.91	−1.99

Free energy of binding (in Kcal/mol) were obtained from AutoDock Vina as the indicator of the binding likelihood. Negative values of free energy indicate possible binding in simulation. More negative scoring indicates stronger binding affinity. ACE, CRP, PTGS, and TNF receptor 2 were not assessable due to the large crystal structure.

## Discussion

### DN, R-6, and the Interaction From a Whole-System Perspective

Eleven clusters of pathophysiology were identified from DN. From a systemic perspective, the complement and coagulation cascades, adipocytokine signaling pathway, TNF signaling pathway, HIF-1 signaling pathway, and AMPK signaling pathway were the key pathways mediating the pathogenesis of DN based on the current evidence from *in vivo* and *in vitro* studies. These pathways are also shared by insulin resistance, rheumatoid arthritis, non-alcoholic fatty liver disease, and various types of infectious diseases and cancers, indicating a possibility in repurposing established therapeutics of these diseases for the DN management.

R-6’s mechanistic action involves modulation of complement, coagulation, angiogenesis (VEGF), oxidative stress (HIF-1), innate and adaptive immunity (T cell, B cell receptor and Fc epsilon RI), cell proliferation and survival (PI3K-Akt, MAPK, Ras, ErbB, neurotrophin, and FoxO), cell adhesion (Rap1 and MAPK), and hormonal (prolactin, estrogen, and thyroid) and metabolism (PPAR, sphingolipid, insulin, and purine) pathways.

From the network inferences, R-6 likely acts on DN via the TNF, NOD-like receptor, HIF-1 and PI3K-Akt signaling pathway, complement and coagulation cascades, and renin–angiotensin system. These share similar mechanisms with non-alcoholic fatty liver disease, inflammatory bowel disease, and rheumatoid arthritis, which could support the use of R-6 in these conditions.

From the *in silico* binding affinity assessment, alisol (*Alismatis Rhizoma*), 7-o-methyl morroniside (*Corni Fructus*), campesterol (*Dioscoreae Rhizoma*), paeoniflorin (*Moutan Cortex*), beta-amyrin acetate (*Poria*), and catalpol (*Rehmanniae Radix*) have the highest likelihood of interacting with the proteins involved in these pathways.

### TNF Signaling as the Key Mechanism of R-6’s Action on DN

From KEGG enrichment analysis, the TNF signaling pathway was demonstrated as the key signaling involved in the action of R-6 on DN with the highest number of involved pathways. TNF signaling activates multiple pathways (e.g., NF-κB, JNK, MAPK, and PI3K-Akt) via receptors including TNF receptor type I (TNFR1) and TNF receptor type II (TNFR2), leading to apoptosis, necroptosis, inflammatory response, and vascular response. The TNF signaling pathway has been suggested as a therapeutic target for general CKD (Al-Lamki and Mayadas, 2015; Breyer and Susztak, 2016), autoimmune diseases, and cancers (Shaikh et al., 2018; Steeland et al., 2018). Elevated sera TNFR1 and TNFR2 were associated with reduced total filtration surface per glomerulus, podocyte number per glomerulus, filtration slit frequency, fenestrated endothelium, and increased glomerular basement membrane width, foot process width, mesangial fractional volume, and global glomerular sclerosis in diabetic patients with early nephropathy (Pavkov et al., 2016). Previous longitudinal cohorts also demonstrated that higher baseline serum TNFR1 and TNFR2 levels increased the risk of end-stage kidney disease among type 2 diabetes patients (Niewczas et al., 2012; Pavkov et al., 2015).

TNF-α suppression by inhibitor or antibody alleviated albuminuria, macrophage infiltration, and glomerular and tubular injury in STZ-induced diabetic rats and *Ins2* ^*Akita*^ mice (Awad et al., 2015; Cheng et al., 2021). Nevertheless, clinical pan-TNF intervention leads to concerns of the off-target effect (e.g., fever, hypotension, tuberculosis, and malignancy) as the TNF signaling pathway is a master regulator of innate immunity (Steeland et al., 2018), and TNFR1 is expressed widely in most cell types. Unlike TNFR1, which is pro-inflammatory and apoptotic, TNFR2 is more restricted to endothelial, immune, neuronal, and tumor cells and associated with immunomodulatory and anti-inflammatory effects (Steeland et al., 2018; Murakoshi et al., 2020). Targeted TNFR2 inhibition that modulates the TNF signaling homeostasis could alleviate the off-target effect in TNF signaling pathway intervention (Shaikh et al., 2018; Steeland et al., 2018; Murakoshi et al., 2020). The clinical efficacy, specificity to TNFR2, and the associated adverse events of R-6 on DN requires further clinical studies.

### Differences Between R-6—DN Interaction Among R-6 Variations

From the comparative analysis of the interaction between R-6 variations and DN, formulations 1, 3, and 5 involved the NOD-like receptor signaling pathway, and formulations 2 and 4 involved regulation of TRP channels on top of the common targeting mechanisms, respectively. Similar to and synergistic with the toll-like receptor, NOD-like receptors are highly conserved pattern recognition receptors that mediate innate immunity through the activation of NF-κB, MAPK, inflammasome, and the production of pro-inflammatory cytokines and chemokines (Geddes et al., 2009). Polymorphisms of TLR and NLR were also associated with dyslipidemia, higher glucose level (Geddes et al., 2009; Gomes Torres et al., 2019), and insulin resistance (Zhou et al., 2017). NLRP3 has been shown to induce epithelial–mesenchymal transition of the tubular epithelial cells via TGF-beta (Wang et al., 2013). A previous study showed that NOD2 was upregulated in diabetic patient biopsies, and NOD2 knockout alleviated the hyperglycemia-induced nephrin expression reduction in diabetic mice (Du et al., 2013).

TRP channels are extensively expressed in the kidney and pancreas that modulate the transporting and signaling mechanisms underpinning glomerular filtration, reabsorption, and secretion of water and solutes in the kidney, and insulin secretion in the pancreas (Tomilin et al., 2016). TRP channels have six subfamilies, namely, TRPC, TRPV, TRPM, TRPP, TRPA, TRPML, and TRPN (Tomilin et al., 2016). TRP channels respond to mechanical stimuli and mediate vascular remodeling in various disease models (Smani et al., 2015; Kanthakumar and Adebiyi, 2021). Under a diabetic environment, the TRPC6 expression increases via the renin–angiotensin system and ATP signaling, leading to calcium influx and subsequent damage of the podocyte structure and detachment (Roshanravan and Dryer, 2014; Sonneveld et al., 2014; Wang et al., 2020). A late clinical trial TRACTION-2 using GFB-887, a podocyte targeted small molecule TRPC5 inhibitor, to treat diabetic kidney disease was designed based on previous *in vitro* and *in vivo* studies showing a protective effect of TRPC5 inhibition via the Rac1 signaling pathway (Walsh et al., 2021). Goshajinkigan, a Japanese herbal formulation similar to R-6, was shown to suppress the oxaliplatin-induced increase of TRPA1 and TRPM8 mRNA expression in dorsal root ganglia (Mizuno et al., 2014). Nevertheless, the involvement of the NOD-like receptor pathway and TRP channels was revealed through network inferences. Current research on the direct effect of R-6 on these two mechanisms in diabetes is limited.

### Strengths and Limitations

A network pharmacological approach was used to delineate the systemic interaction between DN and R-6 with multifactorial pathophysiology and multiple chemical constituents. The whole-system effect of R-6 specific to DN was revealed for further clinical validation. Nevertheless, we limited the analysis to DN with macroalbuminuria for a more specific DN diagnosis (Persson and Rossing, 2018), and we did not perform HPLC or UPLC analysis on the chemical constituents of the formulations as the CMs involved are well studied and available in the existing databases. Network pharmacology analysis focuses on protein–protein interaction. The direction of effect (e.g., positive, negative, or cyclic effect) and the associated epigenetic regulations require further investigations.

Besides, we did not perform *in vivo* or *in vitro* validation as (1) the study medication involves multiple drugs and chemicals, (2) network pharmacology analysis is already a systematic integration of all existing *in vivo* and *in vitro* data (Hopkins, 2008), and (3) further validation data from a single study with experimental models are unlikely to add much evidence to the result from network analysis. We used *in silico* docking analysis as a validation from the chemistry perspective. Free energy was used to estimate the strength of bonding for better estimating the binding affinity instead of using root-mean-square deviation from geometric assessment (Cournia et al., 2017; Mobley and Gilson, 2017). Subsequent validation from clinical samples screening the biomarkers of key mechanisms would serve better to evaluate the whole-system effect and the relative involvement of different mechanisms of the formulations in real clinical settings (Chan et al., 2021c).

## Conclusion

The complement and coagulation cascades, adipocytokine signaling pathway, TNF signaling pathway, HIF-1 signaling pathway, and AMPK signaling pathway orchestrated the pathogenesis of DN from a systemic perspective. The therapeutic effect of R-6 on DN likely involves the TNF, NOD-like receptor, HIF-1, and PI3K-Akt signaling pathways; the complement and coagulation cascades; and the renin–angiotensin system. The analysis of R-6 and the comparative analysis of R-6 variations converged to suggest that the TNF signaling pathway is the key mechanism involved in the action of R-6 on DN among patients with DN presenting with different clinical phenotypes. Variations of R-6 used in clinical protocols may also involve the NOD-like receptor signaling pathway and TRP channels on top of the common mechanisms from indirect inferences. These putative targets could be validated through further trials with clinical samples.

## Data Availability

The original contributions presented in the study are included in the article/Supplementary Material, further inquiries can be directed to the corresponding author.

## Glossary

ACE: Angiotensin-converting enzyme
AMPK: Adenosine monophosphate-activated protein kinase
APOE: Apolipoprotein E
CCL2: chemokine (C-C motif) ligand 2
CKD: Chronic kidney disease
CM: Chinese medicine
CRP: C-reactive protein
DAVID: Database for Annotation, Visualization and Integrated Discovery
DKD: Diabetic kidney disease
DN: Diabetic nephropathy
EDN1: Endothelin 1
ErbB: Erythroblastic leukemia viral oncogene homolog
ERK1/2: Extracellular signal-regulated protein kinase 1/2
Fc epsilon RI: Fc epsilon receptor I
RI: Fc epsilon receptor I
FN1: Fibronectin 1
FoxO: Forkhead Box O
HGF: Hepatocyte growth factor
HIF: Hypoxia-inducible factor
HPLC: High-performance liquid chromatography
ICAM1: Intercellular adhesion molecule 1
IL10: interleukin 10
IL1B: Interleukin 1 beta
IL6: Interleukin 6
INS: Insulin
Ins2Akita: Heterozygous Akita spontaneous mutation
JNK: c-Jun N-terminal kinase
KEGG: Kyoto Encyclopedia of Genes and Genomes
LEP: Leptin
MAPK: Mitogen-activated protein kinase
MCODE: Molecular complex detection
MMP9: Matrix metallopeptidase 9
NF-kappa B: Nuclear factor-kappa B
NLR: NOD-like receptor
NLRP3: NOD-like receptor protein 3
OMIM: Online Mendelian Inheritance in Man
PI3K: Phosphoinositide 3-kinases
AKT: Protein kinase B
PPAR: Peroxisome proliferator-activated receptor
PTGS2: Prostaglandin-endoperoxide synthase 2
R-6: Rehmannia-6
Rap1: Ras-related protein 1
Ras: Rat sarcoma
RCSB: Research Collaboratory for Structural Bioinformatics
SERPINE1: Serpin family E member 1
STRING: Search Tool for the Retrieval of Interacting Genes/Proteins
STZ: Streptozotocin
TLR: Toll-like receptor
TNF: Tumor necrosis factor
TNFR1/2: Tumor necrosis factor receptor 1 or 2
TRP: Transient receptor potential
TRPA: Transient receptor potential ankyrin
TRPC: Transient receptor potential canonical
TRPM: Transient receptor potential melastatin
TRPML: Transient receptor potential mucolipin
TRPN: Transient receptor potential no mechanoreceptor potential C
TRPP: Transient receptor potential polycystin
TRPV: Transient receptor potential vanilloi
UPLC: Ultra performance liquid chromatography
