# Supplementary material for: Potential Therapeutic Targets of Rehmannia Formulations on Diabetic Nephropathy: A Comparative Network Pharmacology Analysis
Source: Front Pharmacol. 2022 Mar 21;13:794139. doi: 10.3389/fphar.2022.794139 (PMC8977554; doi:10.3389/fphar.2022.794139)
Supplement: Supplementary file 3 [file DataSheet1.docx]

**Potential therapeutic targets of rehmannia formulations on diabetic nephropathy: a comparative network pharmacology analysis**

**Supplementary File**

|  |  | Page |
| --- | --- | --- |
| S1 | Genes related to diabetic nephropathy (Excel) | Excel |
| S2 | Chemical compounds of R-6 variations (Excel) | Excel |
| S3 | Gene ontology terms of clusters of diabetic nephropathy pathogenesis | 2 |
| S4 | KEGG enrichment analysis on the diabetic nephropathy pathogenesis | 5 |
| S5 | Gene ontology terms of R-6 targets | 6 |
| S6 | KEGG enrichment analysis on the putative R-6 targets | 8 |
| S7 | Gene ontology terms of targets of R-6 on diabetic nephropathy | 11 |
| S8 | KEGG enrichment analysis on the putative targets of R-6 on diabetic nephropathy | 13 |
| S9 | Gene ontology terms of targets of R-6 variations on diabetic nephropathy | 15 |
| S10 | KEGG enrichment analysis on the targets of R-6 variations on diabetic nephropathy | 23 |

**S3 Gene ontology terms of clusters of diabetic nephropathy pathogenesis**

| GO Code | Gene ONTOLOGY TERMS | *P*-value | Bonferroni corrected *p*-value |
| --- | --- | --- | --- |
| GO:0001666 | response to hypoxia | 3.83E-21 | 1.16E-17 |
| GO:0006954 | inflammatory response | 3.09E-18 | 9.34E-15 |
| GO:0042593 | glucose homeostasis | 7.39E-15 | 2.25E-11 |
| GO:0042493 | response to drug | 6.85E-14 | 2.07E-10 |
| GO:0045766 | positive regulation of angiogenesis | 1.10E-12 | 3.31E-09 |
| GO:0008217 | regulation of blood pressure | 1.71E-12 | 5.17E-09 |
| GO:0050900 | leukocyte migration | 3.27E-12 | 9.88E-09 |
| GO:0048661 | positive regulation of smooth muscle cell proliferation | 8.11E-12 | 2.45E-08 |
| GO:0014823 | response to activity | 2.59E-11 | 7.84E-08 |
| GO:0007204 | positive regulation of cytosolic calcium ion concentration | 1.55E-10 | 4.68E-07 |
| GO:0006953 | acute-phase response | 1.56E-10 | 4.73E-07 |
| GO:0002576 | platelet degranulation | 1.80E-10 | 5.45E-07 |
| GO:0010628 | positive regulation of gene expression | 4.70E-10 | 1.42E-06 |
| GO:0045429 | positive regulation of nitric oxide biosynthetic process | 5.00E-10 | 1.51E-06 |
| GO:0007568 | aging | 7.01E-10 | 2.12E-06 |
| GO:0051384 | response to glucocorticoid | 4.61E-09 | 1.39E-05 |
| GO:0045944 | positive regulation of transcription from RNA polymerase II promoter | 5.43E-09 | 1.64E-05 |
| GO:0071222 | cellular response to lipopolysaccharide | 6.33E-09 | 1.91E-05 |
| GO:0043066 | negative regulation of apoptotic process | 8.42E-09 | 2.55E-05 |
| GO:0009612 | response to mechanical stimulus | 1.84E-08 | 5.55E-05 |
| GO:0032496 | response to lipopolysaccharide | 2.76E-08 | 8.36E-05 |
| GO:0032869 | cellular response to insulin stimulus | 3.40E-08 | 1.03E-04 |
| GO:0030195 | negative regulation of blood coagulation | 4.77E-08 | 1.44E-04 |
| GO:0043410 | positive regulation of MAPK cascade | 6.10E-08 | 1.84E-04 |
| GO:0050766 | positive regulation of phagocytosis | 6.41E-08 | 1.94E-04 |
| GO:0001525 | angiogenesis | 1.01E-07 | 3.06E-04 |
| GO:0046427 | positive regulation of JAK-STAT cascade | 1.47E-07 | 4.43E-04 |
| GO:0006879 | cellular iron ion homeostasis | 1.60E-07 | 4.83E-04 |
| GO:0043491 | protein kinase B signaling | 1.94E-07 | 5.85E-04 |
| GO:0031100 | organ regeneration | 2.91E-07 | 8.80E-04 |
| GO:0006955 | immune response | 3.11E-07 | 9.39E-04 |
| GO:0090026 | positive regulation of monocyte chemotaxis | 3.87E-07 | 0.001169 |
| GO:0051092 | positive regulation of NF-kappaB transcription factor activity | 3.90E-07 | 0.001179 |
| GO:0070374 | positive regulation of ERK1 and ERK2 cascade | 4.05E-07 | 0.001223 |
| GO:0050728 | negative regulation of inflammatory response | 4.17E-07 | 0.00126 |
| GO:0070328 | triglyceride homeostasis | 5.28E-07 | 0.001594 |
| GO:0050731 | positive regulation of peptidyl-tyrosine phosphorylation | 6.13E-07 | 0.001851 |
| GO:0006508 | proteolysis | 7.86E-07 | 0.002374 |
| GO:0030335 | positive regulation of cell migration | 7.98E-07 | 0.00241 |
| GO:0009749 | response to glucose | 8.39E-07 | 0.002532 |
| GO:2000352 | negative regulation of endothelial cell apoptotic process | 9.18E-07 | 0.002772 |
| GO:0048662 | negative regulation of smooth muscle cell proliferation | 1.19E-06 | 0.003591 |
| GO:0046627 | negative regulation of insulin receptor signaling pathway | 1.19E-06 | 0.003591 |
| GO:0045909 | positive regulation of vasodilation | 1.19E-06 | 0.003591 |
| GO:0034374 | low-density lipoprotein particle remodeling | 1.23E-06 | 0.003707 |
| GO:0071260 | cellular response to mechanical stimulus | 1.26E-06 | 0.003814 |
| GO:0007565 | female pregnancy | 1.41E-06 | 0.004263 |
| GO:0019229 | regulation of vasoconstriction | 1.75E-06 | 0.005281 |
| GO:0071356 | cellular response to tumor necrosis factor | 1.82E-06 | 0.005478 |
| GO:0031667 | response to nutrient levels | 1.94E-06 | 0.005842 |
| GO:0007165 | signal transduction | 2.12E-06 | 0.006397 |
| GO:0071407 | cellular response to organic cyclic compound | 2.18E-06 | 0.006573 |
| GO:0050873 | brown fat cell differentiation | 2.44E-06 | 0.007346 |
| GO:0031663 | lipopolysaccharide-mediated signaling pathway | 2.44E-06 | 0.007346 |
| GO:0032755 | positive regulation of interleukin-6 production | 2.45E-06 | 0.007366 |
| GO:0044267 | cellular protein metabolic process | 3.83E-06 | 0.011503 |
| GO:0008285 | negative regulation of cell proliferation | 4.28E-06 | 0.012861 |
| GO:0014068 | positive regulation of phosphatidylinositol 3-kinase signaling | 5.00E-06 | 0.015005 |
| GO:0045087 | innate immune response | 5.22E-06 | 0.015643 |
| GO:0032868 | response to insulin | 6.46E-06 | 0.019344 |
| GO:0050796 | regulation of insulin secretion | 6.46E-06 | 0.019344 |
| GO:0008284 | positive regulation of cell proliferation | 6.63E-06 | 0.019853 |
| GO:0050830 | defense response to Gram-positive bacterium | 6.71E-06 | 0.020073 |
| GO:0008203 | cholesterol metabolic process | 7.32E-06 | 0.021883 |
| GO:0010888 | negative regulation of lipid storage | 9.87E-06 | 0.029387 |
| GO:0000302 | response to reactive oxygen species | 9.90E-06 | 0.029476 |
| GO:0071347 | cellular response to interleukin-1 | 1.05E-05 | 0.031246 |
| GO:0044130 | negative regulation of growth of symbiont in host | 1.07E-05 | 0.031842 |
| GO:0002407 | dendritic cell chemotaxis | 1.49E-05 | 0.0441 |
| GO:0030168 | platelet activation | 1.74E-05 | 0.05137 |
| GO:0032930 | positive regulation of superoxide anion generation | 1.75E-05 | 0.05148 |
|  |  |  |  |

**S4 KEGG enrichment analysis on the diabetic nephropathy pathogenesis**

| Functions | Genes | *P*-value | Bonferroni corrected *p*-value |
| --- | --- | --- | --- |
| hsa05144 | Malaria | 1.74E-09 | 4.01E-07 |
| hsa04610 | Complement and coagulation cascades | 1.94E-09 | 4.49E-07 |
| hsa05142 | Chagas disease (American trypanosomiasis) | 1.59E-08 | 3.67E-06 |
| hsa04920 | Adipocytokine signaling pathway | 2.15E-08 | 4.97E-06 |
| hsa04668 | TNF signaling pathway | 2.47E-08 | 5.72E-06 |
| hsa04066 | HIF-1 signaling pathway | 3.22E-08 | 7.43E-06 |
| hsa05143 | African trypanosomiasis | 3.41E-08 | 7.87E-06 |
| hsa05200 | Pathways in cancer | 3.69E-06 | 8.53E-04 |
| hsa04932 | Non-alcoholic fatty liver disease (NAFLD) | 1.63E-05 | 0.003764 |
| hsa04060 | Cytokine-cytokine receptor interaction | 1.91E-05 | 0.004394 |
| hsa04931 | Insulin resistance | 2.69E-05 | 0.006185 |
| hsa05020 | Prion diseases | 6.72E-05 | 0.015403 |
| hsa05323 | Rheumatoid arthritis | 7.97E-05 | 0.01824 |
| hsa05133 | Pertussis | 9.73E-05 | 0.022219 |
| hsa04152 | AMPK signaling pathway | 1.06E-04 | 0.024218 |
|  |  |  |  |

**S5 Gene ontology terms of R-6 targets**

| GO Code | Gene ONTOLOGY TERMS | *P*-value | Bonferroni corrected *p*-value |
| --- | --- | --- | --- |
| GO:0043401 | steroid hormone mediated signaling pathway | 5.34E-22 | 1.72E-18 |
| GO:0038083 | peptidyl-tyrosine autophosphorylation | 1.28E-15 | 4.29E-12 |
| GO:0006367 | transcription initiation from RNA polymerase II promoter | 2.83E-15 | 9.29E-12 |
| GO:0043066 | negative regulation of apoptotic process | 1.58E-14 | 5.07E-11 |
| GO:0006508 | proteolysis | 2.70E-14 | 8.68E-11 |
| GO:0046777 | protein autophosphorylation | 6.62E-14 | 2.13E-10 |
| GO:0022617 | extracellular matrix disassembly | 8.80E-12 | 2.83E-08 |
| GO:0018108 | peptidyl-tyrosine phosphorylation | 8.47E-11 | 2.73E-07 |
| GO:0001666 | response to hypoxia | 1.41E-10 | 4.53E-07 |
| GO:0055114 | oxidation-reduction process | 4.77E-10 | 1.53E-06 |
| GO:0042493 | response to drug | 4.79E-10 | 1.54E-06 |
| GO:0018105 | peptidyl-serine phosphorylation | 6.34E-10 | 2.04E-06 |
| GO:0014068 | positive regulation of phosphatidylinositol 3-kinase signaling | 1.06E-09 | 3.40E-06 |
| GO:0006468 | protein phosphorylation | 1.15E-09 | 3.71E-06 |
| GO:0032869 | cellular response to insulin stimulus | 1.19E-09 | 3.82E-06 |
| GO:0006749 | glutathione metabolic process | 1.56E-09 | 5.02E-06 |
| GO:0048015 | phosphatidylinositol-mediated signaling | 2.18E-09 | 7.03E-06 |
| GO:0030574 | collagen catabolic process | 8.97E-09 | 2.89E-05 |
| GO:0043627 | response to estrogen | 1.09E-08 | 3.52E-05 |
| GO:0050900 | leukocyte migration | 1.96E-08 | 6.30E-05 |
| GO:0043101 | purine-containing compound salvage | 2.37E-08 | 7.62E-05 |
| GO:0008284 | positive regulation of cell proliferation | 2.40E-08 | 7.71E-05 |
| GO:0007165 | signal transduction | 2.60E-08 | 8.38E-05 |
| GO:0007169 | transmembrane receptor protein tyrosine kinase signaling pathway | 2.79E-08 | 8.99E-05 |
| GO:0030522 | intracellular receptor signaling pathway | 3.38E-08 | 1.09E-04 |
| GO:0018107 | peptidyl-threonine phosphorylation | 3.38E-08 | 1.09E-04 |
| GO:1901687 | glutathione derivative biosynthetic process | 4.83E-08 | 1.56E-04 |
| GO:0030168 | platelet activation | 5.24E-08 | 1.69E-04 |
| GO:0030335 | positive regulation of cell migration | 8.10E-08 | 2.61E-04 |
| GO:0046686 | response to cadmium ion | 1.52E-07 | 4.91E-04 |
| GO:0007173 | epidermal growth factor receptor signaling pathway | 1.82E-07 | 5.85E-04 |
| GO:0048661 | positive regulation of smooth muscle cell proliferation | 3.80E-07 | 0.001224 |
| GO:0001525 | angiogenesis | 4.44E-07 | 0.001427 |
| GO:0006809 | nitric oxide biosynthetic process | 4.59E-07 | 0.001477 |
| GO:0045471 | response to ethanol | 6.11E-07 | 0.001965 |
| GO:0070374 | positive regulation of ERK1 and ERK2 cascade | 8.21E-07 | 0.00264 |
| GO:0001934 | positive regulation of protein phosphorylation | 1.19E-06 | 0.003813 |
| GO:0006006 | glucose metabolic process | 1.21E-06 | 0.003887 |
| GO:0000165 | MAPK cascade | 1.66E-06 | 0.005328 |
| GO:0010628 | positive regulation of gene expression | 1.66E-06 | 0.005328 |
| GO:0007596 | blood coagulation | 1.71E-06 | 0.005483 |
| GO:0042127 | regulation of cell proliferation | 1.85E-06 | 0.005929 |
| GO:0048010 | vascular endothelial growth factor receptor signaling pathway | 2.53E-06 | 0.00811 |
| GO:0043406 | positive regulation of MAP kinase activity | 2.74E-06 | 0.008795 |
| GO:0031100 | organ regeneration | 3.02E-06 | 0.009679 |
| GO:0051289 | protein homotetramerization | 3.22E-06 | 0.010302 |
| GO:0048384 | retinoic acid receptor signaling pathway | 4.44E-06 | 0.014204 |
| GO:0002003 | angiotensin maturation | 4.90E-06 | 0.01564 |
| GO:0014066 | regulation of phosphatidylinositol 3-kinase signaling | 5.65E-06 | 0.018036 |
| GO:0051384 | response to glucocorticoid | 6.80E-06 | 0.021639 |
| GO:0071222 | cellular response to lipopolysaccharide | 8.18E-06 | 0.02599 |
| GO:0043154 | negative regulation of cysteine-type endopeptidase activity involved in apoptotic process | 1.18E-05 | 0.037143 |
| GO:0044267 | cellular protein metabolic process | 1.32E-05 | 0.041499 |
| GO:0008202 | steroid metabolic process | 1.35E-05 | 0.042575 |
| GO:0005975 | carbohydrate metabolic process | 1.41E-05 | 0.044421 |
| GO:0048013 | ephrin receptor signaling pathway | 1.47E-05 | 0.046329 |

**S6 KEGG enrichment analysis on the putative R-6 targets**

| GO Code | Gene ONTOLOGY TERMS | *P*-value | Bonferroni corrected *p*-value |
| --- | --- | --- | --- |
| hsa05200 | Pathways in cancer | 5.55E-13 | 1.49E-10 |
| hsa01100 | Metabolic pathways | 2.24E-11 | 6.04E-09 |
| hsa05205 | Proteoglycans in cancer | 3.28E-11 | 8.83E-09 |
| hsa04014 | Ras signaling pathway | 7.49E-11 | 2.01E-08 |
| hsa04068 | FoxO signaling pathway | 1.56E-10 | 4.21E-08 |
| hsa01130 | Biosynthesis of antibiotics | 1.84E-10 | 4.96E-08 |
| hsa05212 | Pancreatic cancer | 3.64E-10 | 9.79E-08 |
| hsa04917 | Prolactin signaling pathway | 1.92E-09 | 5.16E-07 |
| hsa04914 | Progesterone-mediated oocyte maturation | 2.09E-09 | 5.62E-07 |
| hsa05215 | Prostate cancer | 2.62E-09 | 7.04E-07 |
| hsa04370 | VEGF signaling pathway | 7.07E-09 | 1.90E-06 |
| hsa05210 | Colorectal cancer | 9.28E-09 | 2.50E-06 |
| hsa05223 | Non-small cell lung cancer | 1.33E-08 | 3.59E-06 |
| hsa04915 | Estrogen signaling pathway | 2.49E-08 | 6.71E-06 |
| hsa05211 | Renal cell carcinoma | 2.60E-08 | 7.00E-06 |
| hsa04660 | T cell receptor signaling pathway | 3.01E-08 | 8.09E-06 |
| hsa04910 | Insulin signaling pathway | 3.35E-08 | 9.01E-06 |
| hsa04664 | Fc epsilon RI signaling pathway | 4.22E-08 | 1.14E-05 |
| hsa04012 | ErbB signaling pathway | 7.15E-08 | 1.92E-05 |
| hsa05218 | Melanoma | 8.43E-08 | 2.27E-05 |
| hsa05220 | Chronic myeloid leukemia | 1.05E-07 | 2.83E-05 |
| hsa05230 | Central carbon metabolism in cancer | 1.06E-07 | 2.86E-05 |
| hsa04015 | Rap1 signaling pathway | 1.08E-07 | 2.92E-05 |
| hsa04510 | Focal adhesion | 2.38E-07 | 6.41E-05 |
| hsa04151 | PI3K-Akt signaling pathway | 4.48E-07 | 1.21E-04 |
| hsa04520 | Adherens junction | 4.98E-07 | 1.34E-04 |
| hsa05219 | Bladder cancer | 6.79E-07 | 1.83E-04 |
| hsa04722 | Neurotrophin signaling pathway | 7.88E-07 | 2.12E-04 |
| hsa03320 | PPAR signaling pathway | 1.25E-06 | 3.35E-04 |
| hsa04919 | Thyroid hormone signaling pathway | 1.60E-06 | 4.31E-04 |
| hsa05213 | Endometrial cancer | 1.70E-06 | 4.58E-04 |
| hsa05204 | Chemical carcinogenesis | 2.72E-06 | 7.33E-04 |
| hsa05221 | Acute myeloid leukemia | 4.19E-06 | 0.001126 |
| hsa05214 | Glioma | 4.62E-06 | 0.001241 |
| hsa00980 | Metabolism of xenobiotics by cytochrome P450 | 4.70E-06 | 0.001264 |
| hsa04931 | Insulin resistance | 9.96E-06 | 0.002676 |
| hsa04380 | Osteoclast differentiation | 1.26E-05 | 0.003381 |
| hsa05145 | Toxoplasmosis | 1.30E-05 | 0.00348 |
| hsa05222 | Small cell lung cancer | 2.74E-05 | 0.007343 |
| hsa04066 | HIF-1 signaling pathway | 3.11E-05 | 0.008329 |
| hsa04062 | Chemokine signaling pathway | 3.22E-05 | 0.008627 |
| hsa05120 | Epithelial cell signaling in Helicobacter pylori infection | 3.33E-05 | 0.008925 |
| hsa00982 | Drug metabolism - cytochrome P450 | 3.93E-05 | 0.010514 |
| hsa05152 | Tuberculosis | 4.21E-05 | 0.011256 |
| hsa04610 | Complement and coagulation cascades | 4.62E-05 | 0.012342 |
| hsa04662 | B cell receptor signaling pathway | 4.62E-05 | 0.012342 |
| hsa04010 | MAPK signaling pathway | 4.69E-05 | 0.012543 |
| hsa05160 | Hepatitis C | 5.37E-05 | 0.014337 |
| hsa05161 | Hepatitis B | 5.71E-05 | 0.015239 |
| hsa05231 | Choline metabolism in cancer | 5.91E-05 | 0.015774 |
| hsa00230 | Purine metabolism | 1.11E-04 | 0.029471 |
| hsa04071 | Sphingolipid signaling pathway | 1.47E-04 | 0.038774 |
| hsa00010 | Glycolysis / Gluconeogenesis | 1.50E-04 | 0.039664 |
| hsa05162 | Measles | 1.71E-04 | 0.044978 |

**
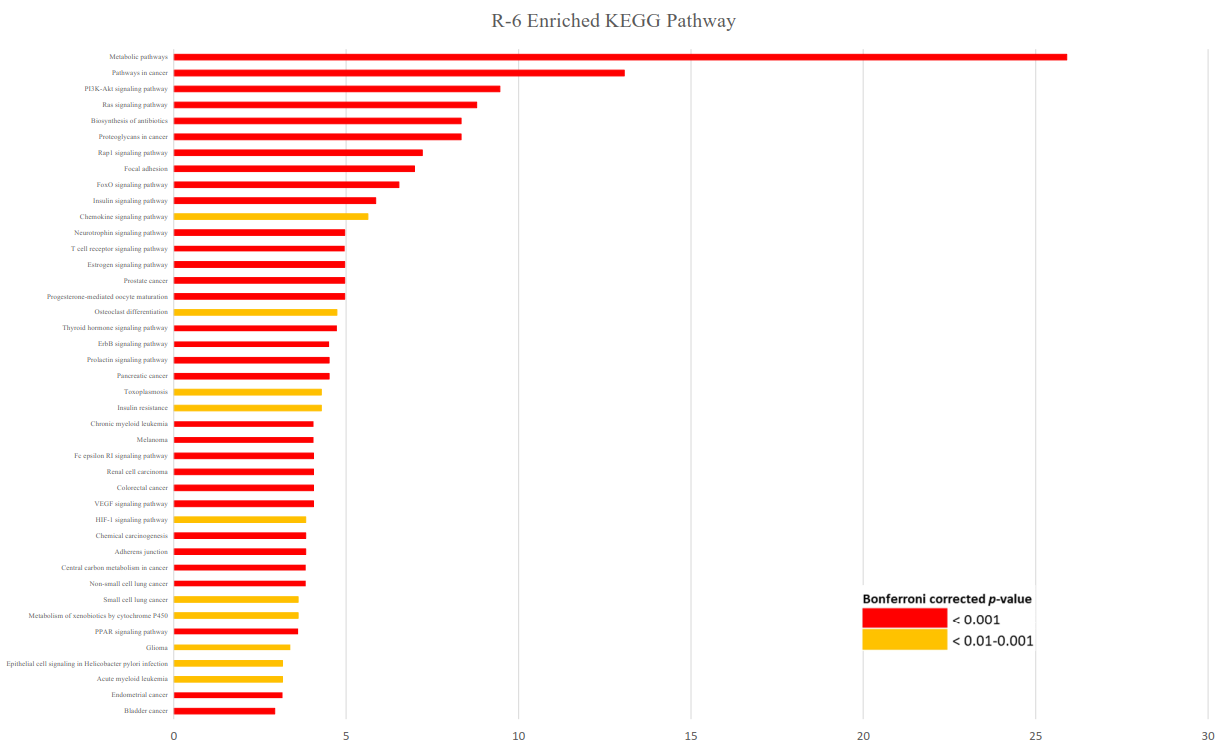
**

**S7 Gene ontology terms of targets of R-6 on diabetic nephropathy**

| GO Code | Gene ONTOLOGY TERMS | *P*-value | Bonferroni corrected *p*-value |
| --- | --- | --- | --- |
|  | **Cluster 1** |  |  |
| GO:0045429 | positive regulation of nitric oxide biosynthetic process | 2.38E-12 | 1.84E-09 |
| GO:0010888 | negative regulation of lipid storage | 4.81E-08 | 3.73E-05 |
| GO:0071222 | cellular response to lipopolysaccharide | 7.32E-08 | 5.68E-05 |
| GO:0006954 | inflammatory response | 1.27E-06 | 9.88E-04 |
| GO:0051091 | positive regulation of sequence-specific DNA binding transcription factor activity | 3.23E-06 | 0.002501 |
| GO:0043200 | response to amino acid | 3.81E-06 | 0.00295 |
| GO:0010628 | positive regulation of gene expression | 4.72E-06 | 0.003659 |
| GO:0006953 | acute-phase response | 7.70E-06 | 0.005958 |
| GO:0051092 | positive regulation of NF-kappaB transcription factor activity | 8.26E-06 | 0.006392 |
| GO:0031622 | positive regulation of fever generation | 9.63E-06 | 0.007445 |
| GO:0014823 | response to activity | 1.04E-05 | 0.008017 |
| GO:0048661 | positive regulation of smooth muscle cell proliferation | 2.85E-05 | 0.021843 |
| GO:0008217 | regulation of blood pressure | 3.62E-05 | 0.027711 |
| GO:0051384 | response to glucocorticoid | 3.62E-05 | 0.027711 |
| GO:0071347 | cellular response to interleukin-1 | 4.72E-05 | 0.035971 |
| GO:0034116 | positive regulation of heterotypic cell-cell adhesion | 5.28E-05 | 0.040127 |
| GO:0001525 | Angiogenesis | 6.29E-05 | 0.047646 |
| GO:0030195 | negative regulation of blood coagulation | 6.33E-05 | 0.047929 |
|  | **Cluster 2** |  |  |
| GO:0014068 | positive regulation of phosphatidylinositol 3-kinase signaling | 6.93E-11 | 4.63E-08 |
| GO:0001525 | Angiogenesis | 2.92E-09 | 1.95E-06 |
| GO:0045766 | positive regulation of angiogenesis | 1.49E-07 | 9.95E-05 |
| GO:0032270 | positive regulation of cellular protein metabolic process | 5.54E-07 | 3.71E-04 |
| GO:0001666 | response to hypoxia | 1.10E-06 | 7.36E-04 |
| GO:0050731 | positive regulation of peptidyl-tyrosine phosphorylation | 1.94E-06 | 0.001294 |
| GO:0002576 | platelet degranulation | 4.82E-06 | 0.00322 |
| GO:0002018 | renin-angiotensin regulation of aldosterone production | 7.27E-06 | 0.00485 |
| GO:0070374 | positive regulation of ERK1 and ERK2 cascade | 3.91E-05 | 0.025815 |
| GO:0030335 | positive regulation of cell migration | 4.76E-05 | 0.031311 |
| GO:0008217 | regulation of blood pressure | 5.13E-05 | 0.03375 |
| GO:0009749 | response to glucose | 5.88E-05 | 0.038545 |
|  | **Cluster 3** |  |  |
| GO:0006954 | inflammatory response | 3.77E-06 | 0.001461 |
| GO:0050900 | leukocyte migration | 1.17E-05 | 0.004546 |
| GO:0032496 | response to lipopolysaccharide | 3.76E-05 | 0.014488 |
|  | **Cluster 4** |  |  |
| GO:2001200 | positive regulation of dendritic cell differentiation | 2.23E-05 | 0.011033 |
| GO:0006954 | inflammatory response | 8.61E-05 | 0.041997 |
|  | **Cluster 5** |  |  |
| GO:0007204 | positive regulation of cytosolic calcium ion concentration | 1.89E-04 | 0.010882 |
|  | **Cluster 6** |  |  |
| GO:0006956 | complement activation | 1.34E-07 | 1.61E-06 |
| GO:0006958 | complement activation, classical pathway | 1.03E-04 | 0.001233 |
| GO:0045087 | innate immune response | 0.001929 | 0.022909 |
| GO:0006508 | Proteolysis | 0.002602 | 0.030783 |
|  | **Cluster 7** |  |  |
| GO:0006839 | mitochondrial transport | 0.001786 | 0.063995 |
|  | **Cluster 8** |  |  |
| GO:0002003 | angiotensin maturation | 0.00131 | 0.041072 |
|  | **Cluster 9** |  |  |
| GO:0090336 | positive regulation of brown fat cell differentiation | 7.15E-04 | 0.022613 |
|  | **Cluster 13** |  |  |
| GO:0042542 | response to hydrogen peroxide | 4.01E-04 | 0.081455 |

**S8 KEGG enrichment analysis on the putative targets of R-6 on diabetic nephropathy**

| GO Code | Gene ONTOLOGY TERMS | *P*-value | Bonferroni corrected *p*-value |
| --- | --- | --- | --- |
|  | **Cluster 1** |  |  |
| hsa05144 | Malaria | 4.54E-10 | 4.77E-08 |
| hsa04668 | TNF signaling pathway | 1.05E-09 | 1.10E-07 |
| hsa05142 | Chagas disease (American trypanosomiasis) | 4.63E-08 | 4.86E-06 |
| hsa05143 | African trypanosomiasis | 5.77E-07 | 6.06E-05 |
| hsa05323 | Rheumatoid arthritis | 3.07E-05 | 0.003213 |
| hsa05146 | Amoebiasis | 6.38E-05 | 0.006677 |
| hsa04060 | Cytokine-cytokine receptor interaction | 1.19E-04 | 0.012371 |
| hsa04621 | NOD-like receptor signaling pathway | 2.17E-04 | 0.022527 |
| hsa04932 | Non-alcoholic fatty liver disease (NAFLD) | 2.52E-04 | 0.026137 |
| hsa05321 | Inflammatory bowel disease (IBD) | 3.23E-04 | 0.033322 |
| hsa05164 | Influenza A | 4.34E-04 | 0.044551 |
| hsa05140 | Leishmaniasis | 4.39E-04 | 0.045023 |
| hsa05144 | Malaria | 4.54E-10 | 4.77E-08 |
| hsa04668 | TNF signaling pathway | 1.05E-09 | 1.10E-07 |
| hsa05142 | Chagas disease (American trypanosomiasis) | 4.63E-08 | 4.86E-06 |
| hsa05143 | African trypanosomiasis | 5.77E-07 | 6.06E-05 |
|  | **Cluster 2** |  |  |
| hsa04066 | HIF-1 signaling pathway | 1.48E-07 | 1.30E-05 |
| hsa05323 | Rheumatoid arthritis | 8.37E-05 | 0.007339 |
| hsa04151 | PI3K-Akt signaling pathway | 2.37E-04 | 0.020633 |
|  | **Cluster 3** |  |  |
| hsa04514 | Cell adhesion molecules (CAMs) | 3.22E-05 | 0.002314 |
|  | **Cluster 5** |  |  |
| hsa05200 | Pathways in cancer | 1.85E-04 | 0.009214 |
| hsa04750 | Inflammatory mediator regulation of TRP channels | 5.97E-04 | 0.029424 |
|  | **Cluster 6** |  |  |
| hsa05150 | Staphylococcus aureus infection | 4.57E-07 | 2.74E-06 |
| hsa04610 | Complement and coagulation cascades | 9.66E-07 | 5.80E-06 |
| hsa05020 | Prion diseases | 7.09E-05 | 4.25E-04 |
| hsa05133 | Pertussis | 3.49E-04 | 0.002095 |
| hsa05142 | Chagas disease (American trypanosomiasis) | 6.72E-04 | 0.004028 |
| hsa05322 | Systemic lupus erythematosus | 0.001116 | 0.006675 |
|  | **Cluster 8** |  |  |
| hsa04614 | Renin-angiotensin system | 0.006676 | 0.032939 |

**S9 Gene ontology terms of targets of R-6 variations on diabetic nephropathy**

| GO Code | Gene ONTOLOGY TERMS | *P*-value | Bonferroni corrected *p*-value |
| --- | --- | --- | --- |
|  | **Formulation 1 Cluster 1** |  |  |
| GO:0045429 | positive regulation of nitric oxide biosynthetic process | 1.54E-12 | 1.18E-09 |
| GO:0010888 | negative regulation of lipid storage | 3.96E-08 | 3.04E-05 |
| GO:0071222 | cellular response to lipopolysaccharide | 5.20E-08 | 3.99E-05 |
| GO:0006954 | inflammatory response | 8.40E-07 | 6.45E-04 |
| GO:0051091 | positive regulation of sequence-specific DNA binding transcription factor activity | 2.48E-06 | 0.001902 |
| GO:0043200 | response to amino acid | 3.14E-06 | 0.002408 |
| GO:0010628 | positive regulation of gene expression | 3.38E-06 | 0.002591 |
| GO:0006953 | acute-phase response | 6.35E-06 | 0.004867 |
| GO:0051092 | positive regulation of NF-kappaB transcription factor activity | 6.36E-06 | 0.004871 |
| GO:0031622 | positive regulation of fever generation | 8.50E-06 | 0.006505 |
| GO:0014823 | response to activity | 8.56E-06 | 0.006551 |
| GO:0048661 | positive regulation of smooth muscle cell proliferation | 2.35E-05 | 0.017884 |
| GO:0008217 | regulation of blood pressure | 2.99E-05 | 0.022706 |
| GO:0051384 | response to glucocorticoid | 2.99E-05 | 0.022706 |
| GO:0071347 | cellular response to interleukin-1 | 3.90E-05 | 0.029504 |
| GO:0034116 | positive regulation of heterotypic cell-cell adhesion | 4.66E-05 | 0.035144 |
| GO:0001525 | angiogenesis | 4.86E-05 | 0.036646 |
| GO:0008284 | positive regulation of cell proliferation | 5.46E-05 | 0.041083 |
| GO:0030195 | negative regulation of blood coagulation | 5.59E-05 | 0.042 |
| GO:0050731 | positive regulation of peptidyl-tyrosine phosphorylation | 6.00E-05 | 0.045062 |
| GO:0051897 | positive regulation of protein kinase B signaling | 6.45E-05 | 0.048344 |
|  | **Formulation 1 Cluster 2** |  |  |
| GO:0014068 | positive regulation of phosphatidylinositol 3-kinase signaling | 6.93E-11 | 4.63E-08 |
| GO:0001525 | angiogenesis | 2.92E-09 | 1.95E-06 |
| GO:0045766 | positive regulation of angiogenesis | 1.49E-07 | 9.95E-05 |
| GO:0032270 | positive regulation of cellular protein metabolic process | 5.54E-07 | 3.71E-04 |
| GO:0001666 | response to hypoxia | 1.10E-06 | 7.36E-04 |
| GO:0050731 | positive regulation of peptidyl-tyrosine phosphorylation | 1.94E-06 | 0.001294 |
| GO:0002576 | platelet degranulation | 4.82E-06 | 0.00322 |
| GO:0002018 | renin-angiotensin regulation of aldosterone production | 7.27E-06 | 0.00485 |
| GO:0070374 | positive regulation of ERK1 and ERK2 cascade | 3.91E-05 | 0.025815 |
| GO:0030335 | positive regulation of cell migration | 4.76E-05 | 0.031311 |
| GO:0008217 | regulation of blood pressure | 5.13E-05 | 0.03375 |
| GO:0009749 | response to glucose | 5.88E-05 | 0.038545 |
|  | **Formulation 1 Cluster 3** |  |  |
| GO:0006954 | inflammatory response | 3.77E-06 | 0.001461 |
| GO:0050900 | leukocyte migration | 1.17E-05 | 0.004546 |
| GO:0032496 | response to lipopolysaccharide | 3.76E-05 | 0.014488 |
|  | **Formulation 1 Cluster 4** |  |  |
| GO:2001200 | positive regulation of dendritic cell differentiation | 2.23E-05 | 0.011033 |
| GO:0006954 | inflammatory response | 8.61E-05 | 0.041997 |
|  | **Formulation 1 Cluster 6** |  |  |
| GO:0006956 | complement activation | 1.34E-07 | 1.61E-06 |
| GO:0006958 | complement activation, classical pathway | 1.03E-04 | 0.001233 |
| GO:0045087 | innate immune response | 0.001929 | 0.022909 |
| GO:0006508 | proteolysis | 0.002602 | 0.030783 |
|  | **Formulation 1 Cluster 8** |  |  |
| GO:0006839 | mitochondrial transport | 0.001786 | 0.063995 |
|  | **Formulation 1 Cluster 9** |  |  |
| GO:0002003 | angiotensin maturation | 0.00131 | 0.041072 |
|  | **Formulation 1 Cluster 10** |  |  |
| GO:0090336 | positive regulation of brown fat cell differentiation | 0.022613 | 7.15E-04 |
|  | **Formulation 1 Cluster 12** |  |  |
| GO:0042542 | response to hydrogen peroxide | 4.01E-04 | 0.081455 |
|  | **Formulation 2 Cluster 1** |  |  |
| GO:0014068 | positive regulation of phosphatidylinositol 3-kinase signaling | 2.12E-14 | 2.58E-11 |
| GO:0001525 | angiogenesis | 6.38E-14 | 7.77E-11 |
| GO:0045429 | positive regulation of nitric oxide biosynthetic process | 6.26E-12 | 7.63E-09 |
| GO:0050731 | positive regulation of peptidyl-tyrosine phosphorylation | 1.24E-11 | 1.51E-08 |
| GO:0008217 | regulation of blood pressure | 1.30E-10 | 1.59E-07 |
| GO:0045766 | positive regulation of angiogenesis | 1.95E-10 | 2.38E-07 |
| GO:0002576 | platelet degranulation | 3.48E-09 | 4.24E-06 |
| GO:0048661 | positive regulation of smooth muscle cell proliferation | 4.79E-09 | 5.84E-06 |
| GO:0001666 | response to hypoxia | 4.83E-09 | 5.89E-06 |
| GO:0070374 | positive regulation of ERK1 and ERK2 cascade | 5.54E-09 | 6.75E-06 |
| GO:0031663 | lipopolysaccharide-mediated signaling pathway | 9.96E-09 | 1.21E-05 |
| GO:0006954 | inflammatory response | 1.08E-08 | 1.32E-05 |
| GO:0043491 | protein kinase B signaling | 1.17E-08 | 1.43E-05 |
| GO:0032270 | positive regulation of cellular protein metabolic process | 3.33E-08 | 4.06E-05 |
| GO:0014823 | response to activity | 4.67E-08 | 5.70E-05 |
| GO:0008284 | positive regulation of cell proliferation | 7.60E-08 | 9.26E-05 |
| GO:0010628 | positive regulation of gene expression | 1.28E-07 | 1.56E-04 |
| GO:0030335 | positive regulation of cell migration | 1.94E-07 | 2.36E-04 |
| GO:0042102 | positive regulation of T cell proliferation | 2.58E-07 | 3.14E-04 |
| GO:0050900 | leukocyte migration | 3.49E-07 | 4.25E-04 |
| GO:2000352 | negative regulation of endothelial cell apoptotic process | 4.89E-07 | 5.95E-04 |
| GO:0051092 | positive regulation of NF-kappaB transcription factor activity | 5.80E-07 | 7.07E-04 |
| GO:0071347 | cellular response to interleukin-1 | 6.03E-07 | 7.35E-04 |
| GO:0010888 | negative regulation of lipid storage | 6.43E-07 | 7.84E-04 |
| GO:0043200 | response to amino acid | 7.47E-07 | 9.10E-04 |
| GO:1902042 | negative regulation of extrinsic apoptotic signaling pathway via death domain receptors | 9.68E-07 | 0.00118 |
| GO:0043410 | positive regulation of MAPK cascade | 1.17E-06 | 0.001421 |
| GO:0051897 | positive regulation of protein kinase B signaling | 1.40E-06 | 0.001704 |
| GO:0007568 | aging | 2.05E-06 | 0.002496 |
| GO:0030195 | negative regulation of blood coagulation | 2.51E-06 | 0.003057 |
| GO:0050901 | leukocyte tethering or rolling | 3.26E-06 | 0.003966 |
| GO:0071356 | cellular response to tumor necrosis factor | 5.31E-06 | 0.006449 |
| GO:0030198 | extracellular matrix organization | 5.55E-06 | 0.006737 |
| GO:0071222 | cellular response to lipopolysaccharide | 6.06E-06 | 0.007355 |
| GO:0071407 | cellular response to organic cyclic compound | 1.03E-05 | 0.012493 |
| GO:0001934 | positive regulation of protein phosphorylation | 1.07E-05 | 0.012974 |
| GO:0019229 | regulation of vasoconstriction | 1.28E-05 | 0.01554 |
| GO:0009611 | response to wounding | 1.34E-05 | 0.016211 |
| GO:0060326 | cell chemotaxis | 1.52E-05 | 0.018345 |
| GO:0051384 | response to glucocorticoid | 1.52E-05 | 0.018345 |
| GO:0046427 | positive regulation of JAK-STAT cascade | 1.73E-05 | 0.020869 |
| GO:0035690 | cellular response to drug | 1.93E-05 | 0.023211 |
| GO:0050729 | positive regulation of inflammatory response | 2.41E-05 | 0.028944 |
| GO:0007267 | cell-cell signaling | 2.42E-05 | 0.029115 |
| GO:0007159 | leukocyte cell-cell adhesion | 2.57E-05 | 0.03086 |
| GO:0000165 | MAPK cascade | 2.89E-05 | 0.034564 |
| GO:0045840 | positive regulation of mitotic nuclear division | 2.90E-05 | 0.03476 |
| GO:0032757 | positive regulation of interleukin-8 production | 2.90E-05 | 0.03476 |
| GO:0002018 | renin-angiotensin regulation of aldosterone production | 3.14E-05 | 0.037606 |
| GO:0010575 | positive regulation of vascular endothelial growth factor production | 3.26E-05 | 0.038958 |
|  | **Formulation 2 Cluster 2** |  |  |
| GO:0014068 | positive regulation of phosphatidylinositol 3-kinase signaling | 6.93E-11 | 4.63E-08 |
| GO:0001525 | angiogenesis | 2.92E-09 | 1.95E-06 |
| GO:0045766 | positive regulation of angiogenesis | 1.49E-07 | 9.95E-05 |
| GO:0032270 | positive regulation of cellular protein metabolic process | 5.54E-07 | 3.71E-04 |
| GO:0001666 | response to hypoxia | 1.10E-06 | 7.36E-04 |
| GO:0050731 | positive regulation of peptidyl-tyrosine phosphorylation | 1.94E-06 | 0.001294 |
| GO:0002576 | platelet degranulation | 4.82E-06 | 0.00322 |
| GO:0002018 | renin-angiotensin regulation of aldosterone production | 7.27E-06 | 0.00485 |
| GO:0070374 | positive regulation of ERK1 and ERK2 cascade | 3.91E-05 | 0.025815 |
| GO:0030335 | positive regulation of cell migration | 4.76E-05 | 0.031311 |
| GO:0008217 | regulation of blood pressure | 5.13E-05 | 0.03375 |
| GO:0009749 | response to glucose | 5.88E-05 | 0.038545 |
|  | **Formulation 2 Cluster 3** |  |  |
| GO:0006954 | inflammatory response | 3.77E-06 | 0.001461 |
| GO:0050900 | leukocyte migration | 1.17E-05 | 0.004546 |
| GO:0032496 | response to lipopolysaccharide | 3.76E-05 | 0.014488 |
|  | **Formulation 2 Cluster 4** |  |  |
| GO:2001200 | positive regulation of dendritic cell differentiation | 2.23E-05 | 0.011033 |
| GO:0006954 | inflammatory response | 8.61E-05 | 0.041997 |
|  | **Formulation 2 Cluster 5** |  |  |
| GO:0007204 | positive regulation of cytosolic calcium ion concentration | 1.89E-04 | 0.010882 |
|  | **Formulation 2 Cluster 6** |  |  |
| GO:0006956 | complement activation | 1.34E-07 | 1.61E-06 |
| GO:0006958 | complement activation, classical pathway | 1.03E-04 | 0.001233 |
| GO:0045087 | innate immune response | 0.001929 | 0.022909 |
| GO:0006508 | proteolysis | 0.002602 | 0.030783 |
|  | **Formulation 3 Cluster 1** |  |  |
| GO:0045429 | positive regulation of nitric oxide biosynthetic process | 1.54E-12 | 1.18E-09 |
| GO:0010888 | negative regulation of lipid storage | 3.96E-08 | 3.04E-05 |
| GO:0071222 | cellular response to lipopolysaccharide | 5.20E-08 | 3.99E-05 |
| GO:0006954 | inflammatory response | 8.40E-07 | 6.45E-04 |
| GO:0051091 | positive regulation of sequence-specific DNA binding transcription factor activity | 2.48E-06 | 0.001902 |
| GO:0043200 | response to amino acid | 3.14E-06 | 0.002408 |
| GO:0010628 | positive regulation of gene expression | 3.38E-06 | 0.002591 |
| GO:0006953 | acute-phase response | 6.35E-06 | 0.004867 |
| GO:0051092 | positive regulation of NF-kappaB transcription factor activity | 6.36E-06 | 0.004871 |
| GO:0031622 | positive regulation of fever generation | 8.50E-06 | 0.006505 |
| GO:0014823 | response to activity | 8.56E-06 | 0.006551 |
| GO:0048661 | positive regulation of smooth muscle cell proliferation | 2.35E-05 | 0.017884 |
| GO:0008217 | regulation of blood pressure | 2.99E-05 | 0.022706 |
| GO:0051384 | response to glucocorticoid | 2.99E-05 | 0.022706 |
| GO:0071347 | cellular response to interleukin-1 | 3.90E-05 | 0.029504 |
| GO:0034116 | positive regulation of heterotypic cell-cell adhesion | 4.66E-05 | 0.035144 |
| GO:0001525 | angiogenesis | 4.86E-05 | 0.036646 |
| GO:0008284 | positive regulation of cell proliferation | 5.46E-05 | 0.041083 |
| GO:0030195 | negative regulation of blood coagulation | 5.59E-05 | 0.042 |
| GO:0050731 | positive regulation of peptidyl-tyrosine phosphorylation | 6.00E-05 | 0.045062 |
| GO:0051897 | positive regulation of protein kinase B signaling | 6.45E-05 | 0.048344 |
|  | **Formulation 3 Cluster 2** |  |  |
| GO:0014068 | positive regulation of phosphatidylinositol 3-kinase signaling | 6.93E-11 | 4.63E-08 |
| GO:0001525 | angiogenesis | 2.92E-09 | 1.95E-06 |
| GO:0045766 | positive regulation of angiogenesis | 1.49E-07 | 9.95E-05 |
| GO:0032270 | positive regulation of cellular protein metabolic process | 5.54E-07 | 3.71E-04 |
| GO:0001666 | response to hypoxia | 1.10E-06 | 7.36E-04 |
| GO:0050731 | positive regulation of peptidyl-tyrosine phosphorylation | 1.94E-06 | 0.001294 |
| GO:0002576 | platelet degranulation | 4.82E-06 | 0.00322 |
| GO:0002018 | renin-angiotensin regulation of aldosterone production | 7.27E-06 | 0.00485 |
| GO:0070374 | positive regulation of ERK1 and ERK2 cascade | 3.91E-05 | 0.025815 |
| GO:0030335 | positive regulation of cell migration | 4.76E-05 | 0.031311 |
| GO:0008217 | regulation of blood pressure | 5.13E-05 | 0.03375 |
| GO:0009749 | response to glucose | 5.88E-05 | 0.038545 |
|  | **Formulation 3 Cluster 3** |  |  |
| GO:0006954 | inflammatory response | 3.77E-06 | 0.001461 |
| GO:0050900 | leukocyte migration | 1.17E-05 | 0.004546 |
| GO:0032496 | response to lipopolysaccharide | 3.76E-05 | 0.014488 |
| GO:0009615 | response to virus | 2.87E-04 | 0.105564 |
|  | **Formulation 3 Cluster 4** |  |  |
| GO:2001200 | positive regulation of dendritic cell differentiation | 2.23E-05 | 0.011033 |
| GO:0006954 | inflammatory response | 8.61E-05 | 0.041997 |
|  | **Formulation 3 Cluster 6** |  |  |
| GO:0007204 | positive regulation of cytosolic calcium ion concentration | 1.89E-04 | 0.010882 |
|  | **Formulation 3 Cluster 7** |  |  |
| GO:0006839 | mitochondrial transport | 0.001786 | 0.066075 |
|  | **Formulation 3 Cluster 8** |  |  |
| GO:0002003 | angiotensin maturation | 0.00131 | 0.041072 |
|  | **Formulation 3 Cluster 9** |  |  |
| GO:0090336 | positive regulation of brown fat cell differentiation | 7.15E-04 | 0.022613 |
|  | **Formulation 3 Cluster 10** |  |  |
| GO:0006956 | complement activation | 2.65E-05 | 2.65E-04 |
| GO:0006958 | complement activation, classical pathway | 3.44E-05 | 3.44E-04 |
| GO:0045087 | innate immune response | 6.54E-04 | 0.006523 |
| GO:0006508 | proteolysis | 8.85E-04 | 0.008814 |
|  | **Formulation 3 Cluster 13** |  |  |
| GO:0042542 | response to hydrogen peroxide | 4.01E-04 | 0.081455 |
| GO:0042493 | response to drug | 6.42E-04 | 0.127202 |
|  | **Formulation 4 Cluster 1** |  |  |
| GO:0045429 | positive regulation of nitric oxide biosynthetic process | 1.54E-12 | 1.18E-09 |
| GO:0010888 | negative regulation of lipid storage | 3.96E-08 | 3.04E-05 |
| GO:0071222 | cellular response to lipopolysaccharide | 5.20E-08 | 3.99E-05 |
| GO:0006954 | inflammatory response | 8.40E-07 | 6.45E-04 |
| GO:0051091 | positive regulation of sequence-specific DNA binding transcription factor activity | 2.48E-06 | 0.001902 |
| GO:0043200 | response to amino acid | 3.14E-06 | 0.002408 |
| GO:0010628 | positive regulation of gene expression | 3.38E-06 | 0.002591 |
| GO:0006953 | acute-phase response | 6.35E-06 | 0.004867 |
| GO:0051092 | positive regulation of NF-kappaB transcription factor activity | 6.36E-06 | 0.004871 |
| GO:0031622 | positive regulation of fever generation | 8.50E-06 | 0.006505 |
| GO:0014823 | response to activity | 8.56E-06 | 0.006551 |
| GO:0048661 | positive regulation of smooth muscle cell proliferation | 2.35E-05 | 0.017884 |
| GO:0008217 | regulation of blood pressure | 2.99E-05 | 0.022706 |
| GO:0051384 | response to glucocorticoid | 2.99E-05 | 0.022706 |
| GO:0071347 | cellular response to interleukin-1 | 3.90E-05 | 0.029504 |
| GO:0034116 | positive regulation of heterotypic cell-cell adhesion | 4.66E-05 | 0.035144 |
| GO:0001525 | angiogenesis | 4.86E-05 | 0.036646 |
| GO:0008284 | positive regulation of cell proliferation | 5.46E-05 | 0.041083 |
| GO:0030195 | negative regulation of blood coagulation | 5.59E-05 | 0.042 |
| GO:0050731 | positive regulation of peptidyl-tyrosine phosphorylation | 6.00E-05 | 0.045062 |
| GO:0051897 | positive regulation of protein kinase B signaling | 6.45E-05 | 0.048344 |
|  | **Formulation 4 Cluster 2** |  |  |
| GO:0014068 | positive regulation of phosphatidylinositol 3-kinase signaling | 6.93E-11 | 4.63E-08 |
| GO:0001525 | angiogenesis | 2.92E-09 | 1.95E-06 |
| GO:0045766 | positive regulation of angiogenesis | 1.49E-07 | 9.95E-05 |
| GO:0032270 | positive regulation of cellular protein metabolic process | 5.54E-07 | 3.71E-04 |
| GO:0001666 | response to hypoxia | 1.10E-06 | 7.36E-04 |
| GO:0050731 | positive regulation of peptidyl-tyrosine phosphorylation | 1.94E-06 | 0.001294 |
| GO:0002576 | platelet degranulation | 4.82E-06 | 0.00322 |
| GO:0002018 | renin-angiotensin regulation of aldosterone production | 7.27E-06 | 0.00485 |
| GO:0070374 | positive regulation of ERK1 and ERK2 cascade | 3.91E-05 | 0.025815 |
| GO:0030335 | positive regulation of cell migration | 4.76E-05 | 0.031311 |
| GO:0008217 | regulation of blood pressure | 5.13E-05 | 0.03375 |
| GO:0009749 | response to glucose | 5.88E-05 | 0.038545 |
|  | **Formulation 4 Cluster 3** |  |  |
| GO:0006954 | inflammatory response | 3.77E-06 | 0.001461 |
| GO:0050900 | leukocyte migration | 1.17E-05 | 0.004546 |
| GO:0032496 | response to lipopolysaccharide | 3.76E-05 | 0.014488 |
|  | **Formulation 4 Cluster 4** |  |  |
| GO:2001200 | positive regulation of dendritic cell differentiation | 2.23E-05 | 0.011033 |
| GO:0006954 | inflammatory response | 8.61E-05 | 0.041997 |
|  | **Formulation 4 Cluster 5** |  |  |
| GO:0007204 | positive regulation of cytosolic calcium ion concentration | 1.89E-04 | 0.010882 |
|  | **Formulation 4 Cluster 6** |  |  |
| GO:0006956 | complement activation | 1.34E-07 | 1.61E-06 |
| GO:0006958 | complement activation, classical pathway | 1.03E-04 | 0.001233 |
| GO:0045087 | innate immune response | 0.001929 | 0.022909 |
| GO:0006508 | proteolysis | 0.002602 | 0.030783 |
|  | **Formulation 4 Cluster 8** |  |  |
| GO:0006839 | mitochondrial transport | 0.001786 | 0.063995 |
|  | **Formulation 5 Cluster 1** |  |  |
| GO:1902042 | negative regulation of extrinsic apoptotic signaling pathway via death domain receptors | 7.27E-05 | 0.028445 |
|  |  |  |  |
|  | **Formulation 5 Cluster 2** |  |  |
| GO:0014068 | positive regulation of phosphatidylinositol 3-kinase signaling | 6.93E-11 | 4.63E-08 |
| GO:0001525 | angiogenesis | 2.92E-09 | 1.95E-06 |
| GO:0045766 | positive regulation of angiogenesis | 1.49E-07 | 9.95E-05 |
| GO:0032270 | positive regulation of cellular protein metabolic process | 5.54E-07 | 3.71E-04 |
| GO:0001666 | response to hypoxia | 1.10E-06 | 7.36E-04 |
| GO:0050731 | positive regulation of peptidyl-tyrosine phosphorylation | 1.94E-06 | 0.001294 |
| GO:0002576 | platelet degranulation | 4.82E-06 | 0.00322 |
| GO:0002018 | renin-angiotensin regulation of aldosterone production | 7.27E-06 | 0.00485 |
| GO:0070374 | positive regulation of ERK1 and ERK2 cascade | 3.91E-05 | 0.025815 |
| GO:0030335 | positive regulation of cell migration | 4.76E-05 | 0.031311 |
| GO:0008217 | regulation of blood pressure | 5.13E-05 | 0.03375 |
| GO:0009749 | response to glucose | 5.88E-05 | 0.038545 |
|  | **Formulation 5 Cluster 3** |  |  |
| GO:0006954 | inflammatory response | 3.77E-06 | 0.001461 |
| GO:0050900 | leukocyte migration | 1.17E-05 | 0.004546 |
| GO:0032496 | response to lipopolysaccharide | 3.76E-05 | 0.014488 |
| GO:0009615 | response to virus | 2.87E-04 | 0.105564 |
|  | **Formulation 5 Cluster 4** |  |  |
| GO:2001200 | positive regulation of dendritic cell differentiation | 2.23E-05 | 0.011033 |
| GO:0006954 | inflammatory response | 8.61E-05 | 0.041997 |
|  | **Formulation 5 Cluster 5** |  |  |
| GO:0007204 | positive regulation of cytosolic calcium ion concentration | 1.89E-04 | 0.010882 |
|  | **Formulation 5 Cluster 6** |  |  |
| GO:0006956 | complement activation | 1.34E-07 | 1.61E-06 |
| GO:0006958 | complement activation, classical pathway | 1.03E-04 | 0.001233 |
| GO:0045087 | innate immune response | 0.001929 | 0.022909 |
| GO:0006508 | proteolysis | 0.002602 | 0.030783 |
|  | **Formulation 5 Cluster 9** |  |  |
| GO:0002003 | angiotensin maturation | 0.00131 | 0.041072 |
|  | **Formulation 5 Cluster 10** |  |  |
| GO:0090336 | positive regulation of brown fat cell differentiation | 7.15E-04 | 0.022613 |

**S10 KEGG enrichment analysis on the targets of R-6 variations on diabetic nephropathy**

| GO Code | Gene ONTOLOGY TERMS | *P*-value | Bonferroni corrected *p*-value |
| --- | --- | --- | --- |
|  | **Formulation 1 Cluster 1** |  |  |
| hsa05144 | Malaria | 4.54E-10 | 4.77E-08 |
| hsa04668 | TNF signaling pathway | 1.05E-09 | 1.10E-07 |
| hsa05142 | Chagas disease (American trypanosomiasis) | 4.63E-08 | 4.86E-06 |
| hsa05143 | African trypanosomiasis | 5.77E-07 | 6.06E-05 |
| hsa05323 | Rheumatoid arthritis | 3.07E-05 | 0.003213 |
| hsa05146 | Amoebiasis | 6.38E-05 | 0.006677 |
| hsa04060 | Cytokine-cytokine receptor interaction | 1.19E-04 | 0.012371 |
| hsa04621 | NOD-like receptor signaling pathway | 2.17E-04 | 0.022527 |
| hsa04932 | Non-alcoholic fatty liver disease (NAFLD) | 2.52E-04 | 0.026137 |
| hsa05321 | Inflammatory bowel disease (IBD) | 3.23E-04 | 0.033322 |
| hsa05164 | Influenza A | 4.34E-04 | 0.044551 |
| hsa05140 | Leishmaniasis | 4.39E-04 | 0.045023 |
|  | **Formulation 1 Cluster 2** |  |  |
| hsa04066 | HIF-1 signaling pathway | 1.48E-07 | 1.30E-05 |
| hsa05323 | Rheumatoid arthritis | 8.37E-05 | 0.007339 |
| hsa04151 | PI3K-Akt signaling pathway | 2.37E-04 | 0.020633 |
|  | **Formulation 1 Cluster 3** |  |  |
| hsa04514 | Cell adhesion molecules (CAMs) | 3.22E-05 | 0.002314 |
|  | **Formulation 1 Cluster 6** |  |  |
| hsa05150 | Staphylococcus aureus infection | 4.57E-07 | 2.74E-06 |
| hsa04610 | Complement and coagulation cascades | 9.66E-07 | 5.80E-06 |
| hsa05020 | Prion diseases | 7.09E-05 | 4.25E-04 |
| hsa05133 | Pertussis | 3.49E-04 | 0.002095 |
| hsa05142 | Chagas disease (American trypanosomiasis) | 6.72E-04 | 0.004028 |
| hsa05322 | Systemic lupus erythematosus | 0.001116 | 0.006675 |
|  | **Formulation 1 Cluster 9** |  |  |
| hsa04614 | Renin-angiotensin system | 0.006676 | 0.032939 |
|  | **Formulation 2 Cluster 1** |  |  |
| hsa04614 | Renin-angiotensin system | 1.13E-04 | 0.011323 |
|  | **Formulation 2 Cluster 2** |  |  |
| hsa04066 | HIF-1 signaling pathway | 1.48E-07 | 1.30E-05 |
| hsa05323 | Rheumatoid arthritis | 8.37E-05 | 0.007339 |
| hsa04151 | PI3K-Akt signaling pathway | 2.37E-04 | 0.020633 |
|  | **Formulation 2 Cluster 3** |  |  |
| hsa04514 | Cell adhesion molecules (CAMs) | 3.22E-05 | 0.002314 |
|  | **Formulation 2 Cluster 5** |  |  |
| hsa05200 | Pathways in cancer | 1.85E-04 | 0.009214 |
| hsa04750 | Inflammatory mediator regulation of TRP channels | 5.97E-04 | 0.029424 |
|  | **Formulation 2 Cluster 6** |  |  |
| hsa05150 | Staphylococcus aureus infection | 4.57E-07 | 2.74E-06 |
| hsa04610 | Complement and coagulation cascades | 9.66E-07 | 5.80E-06 |
| hsa05020 | Prion diseases | 7.09E-05 | 4.25E-04 |
| hsa05133 | Pertussis | 3.49E-04 | 0.002095 |
| hsa05142 | Chagas disease (American trypanosomiasis) | 6.72E-04 | 0.004028 |
| hsa05322 | Systemic lupus erythematosus | 0.001116 | 0.006675 |
|  | **Formulation 3 Cluster 1** |  |  |
| hsa05144 | Malaria | 4.54E-10 | 4.77E-08 |
| hsa04668 | TNF signaling pathway | 1.05E-09 | 1.10E-07 |
| hsa05142 | Chagas disease (American trypanosomiasis) | 4.63E-08 | 4.86E-06 |
| hsa05143 | African trypanosomiasis | 5.77E-07 | 6.06E-05 |
| hsa05323 | Rheumatoid arthritis | 3.07E-05 | 0.003213 |
| hsa05146 | Amoebiasis | 6.38E-05 | 0.006677 |
| hsa04060 | Cytokine-cytokine receptor interaction | 1.19E-04 | 0.012371 |
| hsa04621 | NOD-like receptor signaling pathway | 2.17E-04 | 0.022527 |
| hsa04932 | Non-alcoholic fatty liver disease (NAFLD) | 2.52E-04 | 0.026137 |
| hsa05321 | Inflammatory bowel disease (IBD) | 3.23E-04 | 0.033322 |
| hsa05164 | Influenza A | 4.34E-04 | 0.044551 |
| hsa05140 | Leishmaniasis | 4.39E-04 | 0.045023 |
|  | **Formulation 3 Cluster 2** |  |  |
| hsa04066 | HIF-1 signaling pathway | 1.48E-07 | 1.30E-05 |
| hsa05323 | Rheumatoid arthritis | 8.37E-05 | 0.007339 |
| hsa04151 | PI3K-Akt signaling pathway | 2.37E-04 | 0.020633 |
|  | **Formulation 3 Cluster 3** |  |  |
| hsa04514 | Cell adhesion molecules (CAMs) | 3.22E-05 | 0.002314 |
|  | **Formulation 3 Cluster 6** |  |  |
| hsa05200 | Pathways in cancer | 1.85E-04 | 0.009214 |
| hsa04750 | Inflammatory mediator regulation of TRP channels | 5.97E-04 | 0.029424 |
|  | **Formulation 3 Cluster 8** |  |  |
| hsa04614 | Renin-angiotensin system | 0.006676 | 0.032939 |
|  | **Formulation 3 Cluster 10** |  |  |
| hsa05020 | Prion diseases | 2.37E-05 | 1.42E-04 |
| hsa05150 | Staphylococcus aureus infection | 6.05E-05 | 3.63E-04 |
| hsa04610 | Complement and coagulation cascades | 9.92E-05 | 5.95E-04 |
| hsa05133 | Pertussis | 1.17E-04 | 7.04E-04 |
| hsa05142 | Chagas disease (American trypanosomiasis) | 2.26E-04 | 0.001358 |
| hsa05322 | Systemic lupus erythematosus | 3.77E-04 | 0.002258 |
|  | **Formulation 4 Cluster 1** |  |  |
| hsa04614 | Renin-angiotensin system | 1.13E-04 | 0.011323 |
|  | **Formulation 4 Cluster 2** |  |  |
| hsa04066 | HIF-1 signaling pathway | 1.48E-07 | 1.30E-05 |
| hsa05323 | Rheumatoid arthritis | 8.37E-05 | 0.007339 |
| hsa04151 | PI3K-Akt signaling pathway | 2.37E-04 | 0.020633 |
|  | **Formulation 4 Cluster 3** |  |  |
| hsa04514 | Cell adhesion molecules (CAMs) | 3.22E-05 | 0.002314 |
|  | **Formulation 4 Cluster 5** |  |  |
| hsa05200 | Pathways in cancer | 1.85E-04 | 0.009214 |
| hsa04750 | Inflammatory mediator regulation of TRP channels | 5.97E-04 | 0.029424 |
|  | **Formulation 4 Cluster 6** |  |  |
| hsa05150 | Staphylococcus aureus infection | 4.57E-07 | 2.74E-06 |
| hsa04610 | Complement and coagulation cascades | 9.66E-07 | 5.80E-06 |
| hsa05020 | Prion diseases | 7.09E-05 | 4.25E-04 |
| hsa05133 | Pertussis | 3.49E-04 | 0.002095 |
| hsa05142 | Chagas disease (American trypanosomiasis) | 6.72E-04 | 0.004028 |
| hsa05322 | Systemic lupus erythematosus | 0.001116 | 0.006675 |
|  | **Formulation 5 Cluster 1** |  |  |
| ssc04668 | TNF signaling pathway | 3.18E-08 | 3.31E-06 |
| ssc05144 | Malaria | 3.53E-08 | 3.68E-06 |
| ssc05142 | Chagas disease (American trypanosomiasis) | 1.36E-06 | 1.42E-04 |
| ssc05143 | African trypanosomiasis | 3.67E-05 | 0.003813 |
|  | **Formulation 5 Cluster 2** |  |  |
| hsa04066 | HIF-1 signaling pathway | 1.48E-07 | 1.30E-05 |
| hsa05323 | Rheumatoid arthritis | 8.37E-05 | 0.007339 |
| hsa04151 | PI3K-Akt signaling pathway | 2.37E-04 | 0.020633 |
|  | **Formulation 5 Cluster 3** |  |  |
| hsa04514 | Cell adhesion molecules (CAMs) | 3.22E-05 | 0.002314 |
|  | **Formulation 5 Cluster 5** |  |  |
| hsa05200 | Pathways in cancer | 1.85E-04 | 0.009214 |
| hsa04750 | Inflammatory mediator regulation of TRP channels | 5.97E-04 | 0.029424 |
|  | **Formulation 5 Cluster 6** |  |  |
| hsa05150 | Staphylococcus aureus infection | 4.57E-07 | 2.74E-06 |
| hsa04610 | Complement and coagulation cascades | 9.66E-07 | 5.80E-06 |
| hsa05020 | Prion diseases | 7.09E-05 | 4.25E-04 |
| hsa05133 | Pertussis | 3.49E-04 | 0.002095 |
| hsa05142 | Chagas disease (American trypanosomiasis) | 6.72E-04 | 0.004028 |
| hsa05322 | Systemic lupus erythematosus | 0.001116 | 0.006675 |
|  | **Formulation 5 Cluster 9** |  |  |
| hsa04614 | Renin-angiotensin system | 0.006676 | 0.032939 |
| hsa05144 | Malaria | 4.54E-10 | 4.77E-08 |
| hsa04668 | TNF signaling pathway | 1.05E-09 | 1.10E-07 |
| hsa05142 | Chagas disease (American trypanosomiasis) | 4.63E-08 | 4.86E-06 |
| hsa05143 | African trypanosomiasis | 5.77E-07 | 6.06E-05 |
| hsa05323 | Rheumatoid arthritis | 3.07E-05 | 0.003213 |
| hsa05146 | Amoebiasis | 6.38E-05 | 0.006677 |
| hsa04060 | Cytokine-cytokine receptor interaction | 1.19E-04 | 0.012371 |
| hsa04621 | NOD-like receptor signaling pathway | 2.17E-04 | 0.022527 |
| hsa04932 | Non-alcoholic fatty liver disease (NAFLD) | 2.52E-04 | 0.026137 |
| hsa05321 | Inflammatory bowel disease (IBD) | 3.23E-04 | 0.033322 |
| hsa05164 | Influenza A | 4.34E-04 | 0.044551 |
| hsa05140 | Leishmaniasis | 4.39E-04 | 0.045023 |
|  | **Formulation 1 Cluster 2** |  |  |
| hsa04066 | HIF-1 signaling pathway | 1.48E-07 | 1.30E-05 |
| hsa05323 | Rheumatoid arthritis | 8.37E-05 | 0.007339 |
| hsa04151 | PI3K-Akt signaling pathway | 2.37E-04 | 0.020633 |
|  | **Formulation 1 Cluster 3** |  |  |
| hsa04514 | Cell adhesion molecules (CAMs) | 3.22E-05 | 0.002314 |
|  | **Formulation 1 Cluster 6** |  |  |
| hsa05150 | Staphylococcus aureus infection | 4.57E-07 | 2.74E-06 |
| hsa04610 | Complement and coagulation cascades | 9.66E-07 | 5.80E-06 |
| hsa05020 | Prion diseases | 7.09E-05 | 4.25E-04 |
| hsa05133 | Pertussis | 3.49E-04 | 0.002095 |
| hsa05142 | Chagas disease (American trypanosomiasis) | 6.72E-04 | 0.004028 |
| hsa05322 | Systemic lupus erythematosus | 0.001116 | 0.006675 |
|  | **Formulation 1 Cluster 9** |  |  |
| hsa04614 | Renin-angiotensin system | 0.006676 | 0.032939 |
|  | **Formulation 2 Cluster 1** |  |  |
| hsa04614 | Renin-angiotensin system | 1.13E-04 | 0.011323 |
|  | **Formulation 2 Cluster 2** |  |  |
| hsa04066 | HIF-1 signaling pathway | 1.48E-07 | 1.30E-05 |
| hsa05323 | Rheumatoid arthritis | 8.37E-05 | 0.007339 |
| hsa04151 | PI3K-Akt signaling pathway | 2.37E-04 | 0.020633 |
|  | **Formulation 2 Cluster 3** |  |  |
| hsa04514 | Cell adhesion molecules (CAMs) | 3.22E-05 | 0.002314 |
|  | **Formulation 2 Cluster 5** |  |  |
| hsa05200 | Pathways in cancer | 1.85E-04 | 0.009214 |
| hsa04750 | Inflammatory mediator regulation of TRP channels | 5.97E-04 | 0.029424 |
|  | **Formulation 2 Cluster 6** |  |  |
| hsa05150 | Staphylococcus aureus infection | 4.57E-07 | 2.74E-06 |
| hsa04610 | Complement and coagulation cascades | 9.66E-07 | 5.80E-06 |
| hsa05020 | Prion diseases | 7.09E-05 | 4.25E-04 |
| hsa05133 | Pertussis | 3.49E-04 | 0.002095 |
| hsa05142 | Chagas disease (American trypanosomiasis) | 6.72E-04 | 0.004028 |
| hsa05322 | Systemic lupus erythematosus | 0.001116 | 0.006675 |
|  | **Formulation 3 Cluster 1** |  |  |
| hsa05144 | Malaria | 4.54E-10 | 4.77E-08 |
| hsa04668 | TNF signaling pathway | 1.05E-09 | 1.10E-07 |
| hsa05142 | Chagas disease (American trypanosomiasis) | 4.63E-08 | 4.86E-06 |
| hsa05143 | African trypanosomiasis | 5.77E-07 | 6.06E-05 |
| hsa05323 | Rheumatoid arthritis | 3.07E-05 | 0.003213 |
| hsa05146 | Amoebiasis | 6.38E-05 | 0.006677 |
| hsa04060 | Cytokine-cytokine receptor interaction | 1.19E-04 | 0.012371 |
| hsa04621 | NOD-like receptor signaling pathway | 2.17E-04 | 0.022527 |
| hsa04932 | Non-alcoholic fatty liver disease (NAFLD) | 2.52E-04 | 0.026137 |
| hsa05321 | Inflammatory bowel disease (IBD) | 3.23E-04 | 0.033322 |
| hsa05164 | Influenza A | 4.34E-04 | 0.044551 |
| hsa05140 | Leishmaniasis | 4.39E-04 | 0.045023 |
|  | **Formulation 3 Cluster 2** |  |  |
| hsa04066 | HIF-1 signaling pathway | 1.48E-07 | 1.30E-05 |
| hsa05323 | Rheumatoid arthritis | 8.37E-05 | 0.007339 |
| hsa04151 | PI3K-Akt signaling pathway | 2.37E-04 | 0.020633 |
|  | **Formulation 3 Cluster 3** |  |  |
| hsa04514 | Cell adhesion molecules (CAMs) | 3.22E-05 | 0.002314 |
|  | **Formulation 3 Cluster 6** |  |  |
| hsa05200 | Pathways in cancer | 1.85E-04 | 0.009214 |
| hsa04750 | Inflammatory mediator regulation of TRP channels | 5.97E-04 | 0.029424 |
|  | **Formulation 3 Cluster 8** |  |  |
| hsa04614 | Renin-angiotensin system | 0.006676 | 0.032939 |
|  | **Formulation 3 Cluster 10** |  |  |
| hsa05020 | Prion diseases | 2.37E-05 | 1.42E-04 |
| hsa05150 | Staphylococcus aureus infection | 6.05E-05 | 3.63E-04 |
| hsa04610 | Complement and coagulation cascades | 9.92E-05 | 5.95E-04 |
| hsa05133 | Pertussis | 1.17E-04 | 7.04E-04 |
| hsa05142 | Chagas disease (American trypanosomiasis) | 2.26E-04 | 0.001358 |
| hsa05322 | Systemic lupus erythematosus | 3.77E-04 | 0.002258 |
|  | **Formulation 4 Cluster 1** |  |  |
| hsa04614 | Renin-angiotensin system | 1.13E-04 | 0.011323 |
|  | **Formulation 4 Cluster 2** |  |  |
| hsa04066 | HIF-1 signaling pathway | 1.48E-07 | 1.30E-05 |
| hsa05323 | Rheumatoid arthritis | 8.37E-05 | 0.007339 |
| hsa04151 | PI3K-Akt signaling pathway | 2.37E-04 | 0.020633 |
|  | **Formulation 4 Cluster 3** |  |  |
| hsa04514 | Cell adhesion molecules (CAMs) | 3.22E-05 | 0.002314 |
|  | **Formulation 4 Cluster 5** |  |  |
| hsa05200 | Pathways in cancer | 1.85E-04 | 0.009214 |
| hsa04750 | Inflammatory mediator regulation of TRP channels | 5.97E-04 | 0.029424 |
|  | **Formulation 4 Cluster 6** |  |  |
| hsa05150 | Staphylococcus aureus infection | 4.57E-07 | 2.74E-06 |
| hsa04610 | Complement and coagulation cascades | 9.66E-07 | 5.80E-06 |
| hsa05020 | Prion diseases | 7.09E-05 | 4.25E-04 |
| hsa05133 | Pertussis | 3.49E-04 | 0.002095 |
| hsa05142 | Chagas disease (American trypanosomiasis) | 6.72E-04 | 0.004028 |
| hsa05322 | Systemic lupus erythematosus | 0.001116 | 0.006675 |
|  | **Formulation 5 Cluster 1** |  |  |
| ssc04668 | TNF signaling pathway | 3.18E-08 | 3.31E-06 |
| ssc05144 | Malaria | 3.53E-08 | 3.68E-06 |
| ssc05142 | Chagas disease (American trypanosomiasis) | 1.36E-06 | 1.42E-04 |
| ssc05143 | African trypanosomiasis | 3.67E-05 | 0.003813 |
|  | **Formulation 5 Cluster 2** |  |  |
| hsa04066 | HIF-1 signaling pathway | 1.48E-07 | 1.30E-05 |
| hsa05323 | Rheumatoid arthritis | 8.37E-05 | 0.007339 |
| hsa04151 | PI3K-Akt signaling pathway | 2.37E-04 | 0.020633 |
|  | **Formulation 5 Cluster 3** |  |  |
| hsa04514 | Cell adhesion molecules (CAMs) | 3.22E-05 | 0.002314 |
|  | **Formulation 5 Cluster 5** |  |  |
| hsa05200 | Pathways in cancer | 1.85E-04 | 0.009214 |
| hsa04750 | Inflammatory mediator regulation of TRP channels | 5.97E-04 | 0.029424 |
|  | **Formulation 5 Cluster 6** |  |  |
| hsa05150 | Staphylococcus aureus infection | 4.57E-07 | 2.74E-06 |
| hsa04610 | Complement and coagulation cascades | 9.66E-07 | 5.80E-06 |
| hsa05020 | Prion diseases | 7.09E-05 | 4.25E-04 |
| hsa05133 | Pertussis | 3.49E-04 | 0.002095 |
| hsa05142 | Chagas disease (American trypanosomiasis) | 6.72E-04 | 0.004028 |
| hsa05322 | Systemic lupus erythematosus | 0.001116 | 0.006675 |
|  | **Formulation 5 Cluster 9** |  |  |
| hsa04614 | Renin-angiotensin system | 0.006676 | 0.032939 |
